# Targeting metabolic vulnerability by combining NAMPT inhibitors and disulfiram for treatment of recurrent ovarian cancer

**DOI:** 10.1038/s41419-025-07672-3

**Published:** 2025-04-25

**Authors:** Kei Kudo, Yoshimi Endo Greer, Daniel R. Crooks, Ye Yang, Jeffrey R. Brender, Teruhiko Yoshida, Brittney S. Harrington, Rahul Kamdar, Soumya Korrapati, Yusuke Shibuya, Leah Henegar, Jeffrey Kopp, Takeo Fujii, Stanley Lipkowitz, Christina M. Annunziata

**Affiliations:** 1 Women’s Malignancies Branch, National Cancer Institute, National Institutes of Health, Bethesda, MD USA; 2 Department of Obstetrics and Gynecology, Division of Gynecologic Oncology, Tohoku University School of Medicine, Miyagi, Japan; 3 Urologic Oncology Branch, National Cancer Institute, National Institutes of Health, Bethesda, MD USA; 4 Clinical Cancer Metabolism Facility, Center for Cancer Research, National Cancer Institute, Bethesda, MD USA; 5 Kidney Disease Section, Kidney Diseases Branch, National Institute of Diabetes and Digestive and Kidney Diseases, National Institutes of Health, Bethesda, MD USA; 6 Karyopharm Therapeutics, Newton, MA USA

**Keywords:** Ovarian cancer, Metabolomics, Apoptosis, Cancer stem cells

## Abstract

Ovarian cancer (OV) has the highest mortality rate among gynecological cancers. As OV progresses, tumor cells spread outside the ovaries to the peritoneal and abdominal cavities, forming cell clusters that float in the ascitic fluid caused by peritonitis carcinomatosa, leading to further dissemination and metastasis. These cell clusters are enriched with cancer stem cells (CSCs) which are responsible for treatment resistance, recurrence, and metastasis. Therefore, targeting CSCs is a potentially effective approach for treating OV. However, understanding how CSCs acquire treatment resistance and identifying targets against CSCs remains challenging. In this study, we demonstrate that 3D-spheroids of OV cell lines exhibit higher stemness than conventional adherent cells. Metabolomics profiling studies have revealed that 3D-spheroids maintain a high-energy state through increased glucose utilization in the citric acid cycle (TCA), efficient nucleotide phosphorylation, and elevated phosphocreatine as an energy buffer. We also found that nicotinamide phosphoribosyltransferase (NAMPT), the rate-limiting enzyme for NAD^+^ production, is highly expressed in OV. Furthermore, the approach based on NAMPT dependence rather than histology found NAMPT to be a potential therapeutic target against CSCs, while also serving as a prognostic indicator in OV. Moreover, we identified a previously unrecognized anti-tumor mechanism whereby disulfiram, an aldehyde dehydrogenase (ALDH) inhibitor, synergistically inhibited mitochondrial function when combined with NAMPT inhibitors - leading to cell cycle arrest in G2/M. Finally, the combination of a NAMPT inhibitor and disulfiram showed significant anti-tumor effects and extended survival in an animal model. Our findings demonstrate the potential of spheroids as a preclinical model for targeting OV CSCs and also indicate that the combination of NAMPT inhibitors and disulfiram is a promising therapeutic strategy to overcome recurrent OV.

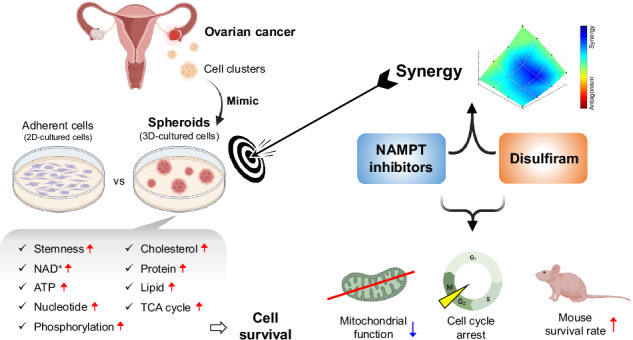

## Introduction

Ovarian cancer (OV) has the highest mortality rate among gynecologic cancers [1, 2]. Although some cases can be cured through multidisciplinary treatment involving surgery and anticancer drugs [3, 4], including angiogenesis inhibitors [5] and poly (ADP-ribose) polymerase (PARP) inhibitors [6], the majority of patients with advanced disease relapse. With an average survival of only 12-18 months post-relapse [7], the dismal prognosis of OV underscores the urgency for innovative treatment strategies.

With disease progression, OV exhibits a distinctive metastatic pattern in which tumor cells extend beyond the ovary, shedding into the peritoneal and abdominal cavities, and forming cell clusters that float in the ascitic fluid caused by peritonitis carcinomatosa, thereby fostering further dissemination and metastasis. These cell clusters are enriched with cancer stem cells (CSCs), which possess unique metabolic properties and contribute to treatment resistance, tumor recurrence, and metastasis [8]. Recently, we optimized preclinical models of OV using 3D-cultured spheroids, which morphologically mimic cell clusters in ascites [9, 10]. Spheroids are enriched in CSCs and reflect the characteristics of recurrent cancer, including tumorigenicity and chemoresistance [11]. We hypothesized that targeting the distinctive metabolism of 3D-cultured spheroids could lead to the development of effective therapies. Using our established technique for culturing uniform 3D-spheroids with ultra-low attachment plates to promote cell suspension, we aimed to explore spheroid characteristics by comparing metabolic differences between 2D- and 3D-cell cultures and identifying strategies for targeting 3D-spheroids.

We found that elevated expression of nicotinamide phosphoribosyltransferase (NAMPT), a key enzyme that converts nicotinamide (NAM) into NAD^+^ [12], represents a potential therapeutic target for OV. Additionally, we demonstrated a robust synergistic effect when combining NAMPT inhibitors with disulfiram, an aldehyde dehydrogenase (ALDH) inhibitor used to treat chronic alcoholism [13, 14]. We further elucidated the mechanism from various perspectives, including metabolomics and the assessment of mitochondrial function. Lastly, the combination of NAMPT inhibitors and disulfiram was confirmed to have a significant anti-tumor effect and extend animal survival. Our findings suggest that this combinatorial treatment could be an effective and novel therapeutic strategy for recurrent OV.

## Results

### NAMPT is a prognostic factor and a potential therapeutic target against 3D-spheroids with cancer stemness features

To morphologically mimic floating cell clusters in the peritoneal cavity of patients with OV [15], we obtained various non-adherent cell lines that proliferated while suspended in the medium using ULA plates (Supplementary Fig. S1A). All the cell lines investigated in this study (A2780, IGROV1, OVCAR3, OVCAR8, ES2, SKOV3) formed 3D-spheroids. We opted to focus on studying the metabolic pathways in A2780 and IGROV1. These cell lines were selected in terms of their dependence on NAMPT for NAD^+^ production, which will be discussed below. To confirm that the spheroids have the characteristics of CSCs functionally and morphologically, we investigated the expression of stem cell markers associated with sphere formation and tumor cell biogenesis using RT-PCR [16, 17]. In the A2780, CD133 and SOX2 were markedly upregulated in 3D-spheroids, while CD44 exhibited a trend toward higher expression in the same condition (Supplementary Fig. S1B). In the IGROV1, most markers did not show significant differences; however, Nanog demonstrated a trend of increased expression in 3D-spheroids (Supplementary Fig. S1B). Additionally, western blot analysis revealed that CD133 expression was significantly elevated in 3D-spheroids in both cell lines (Supplementary Fig. S1C). Also, we measured ALDH activity using the ALDEFLUOR assay. ALDHs play a key role in maintenance of stemness and tumorigenicity in the cancer cell population [18]. Consistent with the above findings, 3D-spheroids in both cell lines exhibited significantly higher mean MFI compared to conventionally cultured 2D cells (Supplementary Fig. S1D). These results suggest that 3D-spheroids obtained using ULA plates are enriched in stemness markers and mimic floating cell clusters in the peritoneal cavity, both morphologically and functionally.

**Fig. 1:**
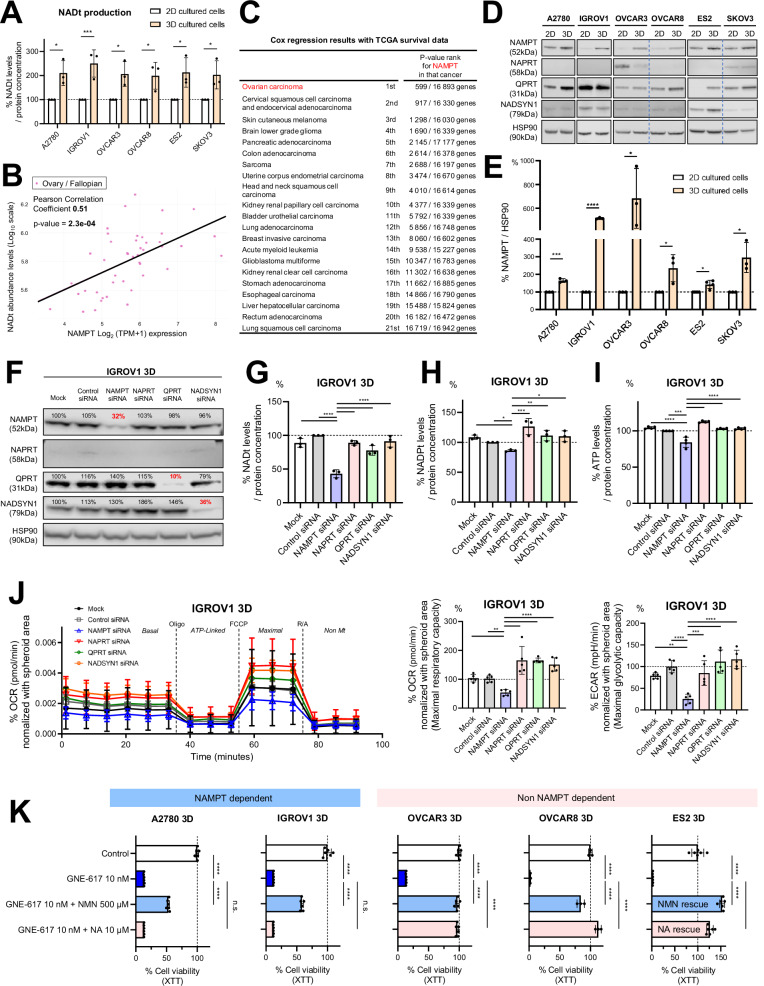
NAMPT is a prognostic factor and a potential therapeutic target against 3D-spheroids with cancer stemness features. **A** Comparison of total NAD levels normalized to protein concentration in 2D- and 3D-cultured cells using A2780, IGROV1, OVCAR3, OVCAR8, ES2, and SKOV3 cells (*n* = 3 independent experiments). **B** Correlations between the gene expression of NAMPT (Public 23Q2) and levels of total NAD metabolites in ovarian or fallopian cancer cell lines, data obtained from the DepMap portal data. **C** *P*-value ranking of NAMPT among approximately 17,000 genes was derived from Cox regression results using TCGA survival data. The rankings were sorted in ascending order for 21 cancer types. **D** Immunoblots to assess the expression of enzymes related to NAD^+^ production in 2D- and 3D-cultured cells using A2780, IGROV1, OVCAR3, OVCAR8, ES2, and SKOV3 cells. HSP90 was used as a control. **E** NAMPT levels normalized to HSP90 in 2D- and 3D-cultured cells using A2780, IGROV1, OVCAR3, OVCAR8, ES2, and SKOV3 cells (*n* = 3 or 4 independent experiments). **F** Immunoblotting to evaluate the silencing of enzymes involved in NAD^+^ production in 3D-cultured IGROV1 cells. HSP90 was used as a control. **G** Comparison of total NAD levels normalized to protein concentration in 3D-cultured IGROV1 cells (*n* = 3 independent experiments). **H** Comparison of ATP levels normalized to protein concentration in 3D-cultured IGROV1 cells (*n* = 3 independent experiments). **I** Comparison of total NADP levels normalized to protein concentration in 3D-cultured IGROV1 cells (*n* = 4 independent experiments). **J** Left: Representative OCR in 3D-cultured IGROV1 cells over time (min), normalized to the spheroid area. Oligomycin (Oligo), carbonyl cyanide-4-(trifluoromethoxy)phenylhydrazone (FCCP), rotenone, and antimycin A (R/A) were added to measure Basal OCR, ATP-Linked OCR, maximal OCR, and non-mitochondrial OCR (*n* = 5 independent experiments). Middle: Maximal respiratory capacity in OCR (*n* = 5 independent experiments). Right: Maximal glycolytic capacity in ECAR (*n* = 5 independent experiments). **K** Relative cell viability after treatment with GNE-617 at the indicated doses for 72 h in 3D-cultured A2780, IGROV1, OVCAR3, OVCAR8, and ES2 cells. NMN or NA was added to the media for rescue experiments (*n* = 3 or 6 independent experiments). Graph data were presented as mean ± SD of multiple experiments.

One of the reasons for the therapy-resistance of CSCs is elevated NAD^+^ production [19]. We hypothesized that 3D-spheroids maintain higher NAD^+^ levels than conventional 2D-cultured cells. In line with this hypothesis, 3D-spheroids exhibited significantly increased NAD^+^ levels compared to 2D-cultured cells across all six cell lines used in this study (Fig. 1A). NAD^+^ is produced via various pathways and acts as co-factors in various reactions [12, 20]. Key enzymes for the NAD^+^ production include NAPRT (Preiss-Handler pathway), QPRT (de novo pathway), NADSYN1 (common to Preiss-Handler and de novo pathways), and NAMPT (salvage pathway) (Supplementary Fig. S1E). We have previously demonstrated that high expression of NAMPT correlates with a substantially reduced overall survival in human ovarian cancer, suggesting that high NAMPT expression could be a prognostic factor in OV [21]. Conversely, there was no statistical difference in the survival of cohorts of patients with OV expressing high or low NAPRT, QPRT, or NADSYN1 (Supplementary Fig. S1F). Furthermore, we evaluated the correlation between NAD^+^ levels and the gene expression of the above-mentioned enzymes in OV cell lines using the public database of the Cancer Dependency Map (DepMap) portal data explorer. Consistent with the overall survival data, NAMPT showed a strong positive correlation with NAD^+^ (Fig. 1B), whereas other enzymes did not show a clear positive correlation (Supplementary Fig. S1G). To better understand the high-risk role of NAMPT, we assessed its prognostic impact in other cancer types. Importantly, OV had the lowest p-value rank, suggesting that elevated NAMPT expression may promote tumor growth by increasing NAD^+^ production in the OV (Fig. 1C). We tested this hypothesis by evaluating NAMPT expression by western blot analysis. NAMPT expression was consistently more abundant in 3D-spheroids than in 2D-cultured cells across all cell lines (Fig. 1D, E), whereas the expression of other enzymes varied depending on cell lines and was not significantly different between 3D-speroids and 2D-cultured cells (Fig. 1D). To investigate the impact of NAMPT silencing on NAD^+^ production, we quantified NAD^+^ levels after individually suppressing enzyme expression using siRNAs. Protein levels of each enzyme were effectively suppressed in both 3D- and 2D-cultured cells (Fig. 1F and Supplementary Fig. S1H), and silencing NAMPT effectively inhibited NAD^+^ production compared to control siRNA, whereas silencing of the other enzymes did not. Also, the two cell lines had barely detectable levels for NAPRT (Fig. 1F, G, Supplementary Fig. S1H, and Supplementary Fig. S1I). Consistent with the known NAD^+^ production pathways, silencing of NAMPT also suppressed NADPH and ATP production (Fig. 1H, I). To determine whether silencing of NAMPT also impaired mitochondrial function, we quantified the oxygen consumption rate (OCR), which is an indicator of mitochondrial respiration. We observed that the OCR of cells with NAMPT siRNA was significantly suppressed in Maximal respiratory capacity as well as in Basal respiration (Fig. 1J and Supplementary Fig. S1J), suggesting that NAMPT is most strongly related to mitochondrial function in this cell line. Silencing NAMPT also reduced the extracellular acidification rate (ECAR), indicating a reduction in glycolytic flux (Fig. 1J). Conversely, suppression of OCR and ECAR was not observed in 2D-cultured cells (Supplementary Fig. S1K).

Notably, the RPMI-1640 medium used in this study contained nicotinamide (NAM) and tryptophan (Trp) but no nicotinic acid (NA). Since human plasma contains NA, we next evaluated the effect of NA on the anti-tumor effect of the NAMPT inhibitor GNE-617 to understand NAD^+^ production kinetics in a more biologically relevant environment (Supplementary Fig. S2A). The addition of NA did not rescue the anti-proliferative effect of NAMPT inhibitors in A2780 and IGROV1 cells, but completely restored the effect in OVCAR3, OVCAR8, and ES2 cells (Fig. 1K and Supplementary Fig. S2B). These results indicate that these latter cell lines can use NAPRT in the Preiss-Handler pathway for NAD^+^ production, whereas A2780 and IGROV1 mainly depend on NAMPT in the salvage pathway (Supplementary Fig. S1E). As expected, the addition of nicotinamide mononucleotide (NMN), a derivative of NAM, rescued the anti-tumor effect of GNE-617 across all cell lines (Fig. 1K), since this molecule is downstream of NAMPT in the salvage pathway (Supplementary Fig. S1E).

Overall, we observed that 3D-spheroids exhibit CSC characteristics with elevated NAD^+^, and NAMPT is the key enzyme that supports NAD^+^ production. These findings suggest that NAMPT inhibition may be potent therapeutic strategy against OV dependent on NAMPT for NAD^+^ production.

### 3D-spheroids oxidize glucose via the TCA cycle for elevated ATP production

**Fig. 2:**
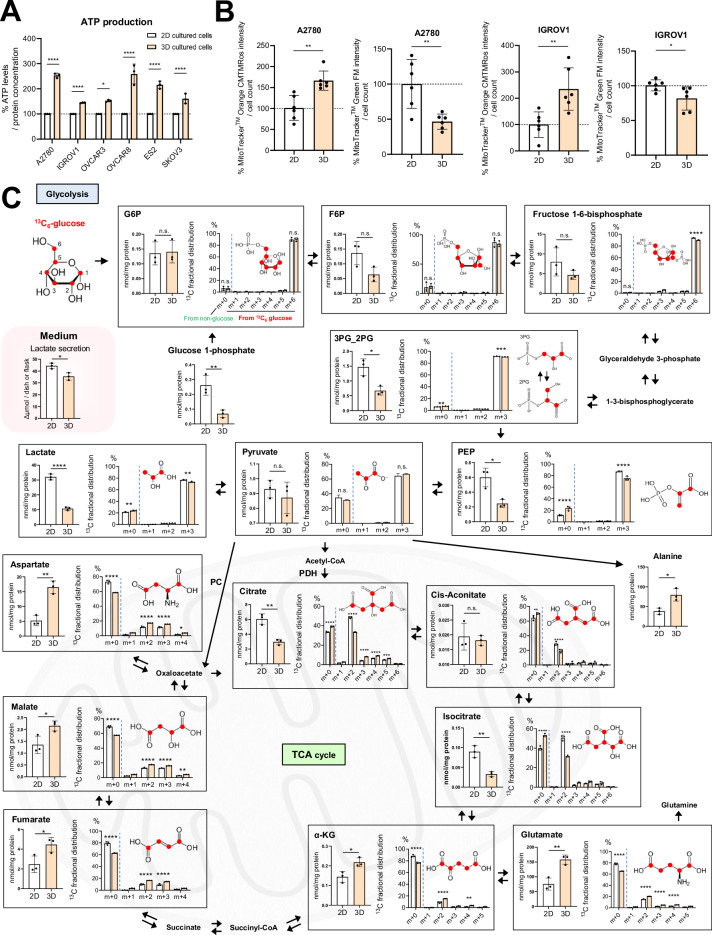
3D-spheroids oxidize glucose via the TCA cycle for elevated ATP production. **A** Comparison of ATP levels normalized to protein concentration in cells cultured in 2D- and 3D-cultured cells using A2780, IGROV1, OVCAR3, OVCAR8, ES2, and SKOV3 cells (*n* = 3 independent experiments). **B** Left: Comparison of MitoTracker Orange CMTMRos and MitoTracker Green FM normalized to cell counts in 2D- and 3D-cultured A2780 cells (*n* = 6 independent experiments). Right: Comparison of MitoTracker Orange CMTMRos and MitoTracker Green FM normalized to cell count in cells cultured in 2D- and 3D-cultured IGROV1 cells (*n* = 6 independent experiments). **C** Comparison of key metabolite levels in glycolysis and TCA cycle in both 2D- and 3D-cultured cells after 24 h of incubation. The carbon distribution of the metabolites was also illustrated. Additionally, lactate secretion into the medium after 24 h of incubation is presented (*n* = 3 independent replicates). The red circles indicate carbon. “m + 0” represents the carbon contribution from sources other than ^13^C_6_-glucose, and “m + 1,” “m + 2,” etc. represent the carbon contribution from ^13^C_6_-glucose (^13^C isotopologues). G6P: glucose-6-phosphate, F6P: fructose-6-phosphate, 3PG_2PG: 3-phosphoglycerate or 2-phosphoglycerate, PEP: phosphoenolpyruvate, α-KG: alpha-ketoglutarate, PC: pyruvate carboxylase, PDH: pyruvate dehydrogenase. Graph data were presented as mean ± SD of multiple experiments.

We observed that the proliferation rate of cells grown in 2D culture was significantly higher than that of cells grown under 3D culture conditions in either 2D- or 3D-medium (Supplementary Fig. S3A). Also, relative viability of 3D-cells was lower than that of 2D in the same 3D-medium (Supplementary Fig. S3B). This difference in proliferation rate may underlie the metabolic differences observed between 2D- and 3D-cultured cells. Given that ATP is produced via glycolysis and the TCA cycle using NAD^+^ as a co-factor [22], we evaluated the total ATP production in 2D- and 3D-cultured cells. Consistent with total NAD (Fig. 1A), we observed more ATP in 3D-spheroids (Fig. 2A). To determine the mechanism of elevated ATP production, we evaluated the function and mass of mitochondria. MitoTracker Orange CMTMRos, which stains mitochondria in living cells in a membrane potential-dependent fashion [22], and MitoTracker Green FM, which is membrane potential-independent and is useful for measuring mitochondrial mass [23], were quantified in A2780 and IGROV1 cells. We observed a significantly higher intensity of MitoTracker Orange CMTMRos in both 3D-cultured cell lines (Fig. 2B). In contrast, MitoTracker Green FM was significantly lower in 3D-spheroids (Fig. 2B). This suggests that mitochondria in 3D-spheroids possess higher ATP production capacity.

We investigated whether the higher ATP production in 3D-spheroids was a result of modified central carbon metabolism. To this end, we used IGROV1 cells, which rely on glucose for growth (Supplementary Fig. S3C). The cells were cultured in a tracer medium containing ^13^C_6_-glucose to investigate the fate of glucose in central metabolic pathways. We evaluated ^13^C_6_-glucose utilization in 2D- and 3D-cultured IGROV1 cells by NMR and ion chromatography-mass spectrometry (IC-MS) (Supplementary Fig. S3D). ^1^H-^13^C HSQC NMR analysis revealed that 3D-cultured cells exhibited increased biosynthesis of glutamine, glutamate, and glutathione from ^13^C_6_-glucose, indicating the enhanced entry and cycling of glucose in the TCA cycle (Supplementary Fig. S3E). Furthermore, resonances arising from de novo-synthesized purine nucleotides (AXP) and components of the hexosamine pathway (e.g., UDP-GlcNAc, UDP-GalNAc, GlcNAc-1-P), which contribute to resistance of cancer stem cells to chemotherapy [24, 25], indicate enhanced distribution of glycolytic intermediates in the pentose phosphate pathway (via glucose-6-phosphate (G6P)) and hexosamine biosynthetic pathway (via fructose-6-phosphate (F6P)). 2D-cultured cells demonstrated ^13^C_6_-glucose labeling of lactate, which exceeded that of alanine, whereas in 3D-cultured cells, ^13^C_6_-glucose labeling of alanine was in excess of that of lactate (Supplementary Fig. S3F), suggesting a shift away from lactate fermentation in the 3D-cultured cells. Although there was no significant difference in the glucose consumption in the culture medium (Supplementary Fig. S3G), 3D-cultured cells exhibited decreased intracellular ^13^C-glucose/G6P, which is consistent with the enhanced entry and distribution of ^13^C-glucose into the pathways outlined above (Supplementary Fig. S3E). Furthermore, the protein levels of hexokinase 2, known for its upregulated expression in cancer cells [26], were significantly higher in 3D-cultured cells (Supplementary Fig. S3H).

IC-MS of the polar extracts revealed no significant differences between 2D- and 3D-cultured cells in the abundance or isotopological distribution of the glycolytic intermediates G6P, F6P, and pyruvate (Fig. 2C). Conversely, 3- and 2-phosphoglycerate (3PG_2PG), phosphoenolpyruvate (PEP), and lactate levels were higher in 2D-cultured cells (Fig. 2C). Furthermore, more lactate was secreted into the culture medium of the 2D-cultures, consistent with elevated lactate fermentation in 2D-cultured cells compared to 3D-cultured cells (Fig. 2C). m + 3 isotopologues of citrate were significantly elevated in 3D-spheroids (Fig. 2C), consistent with pyruvate carboxylase (PC)-mediated entry of glucose-derived carbon into TCA cycle [27, 28] (Supplementary Fig. S4A and S4B) and elevated m + 4 and m + 5 citrate isotopologues indicated multiple turns of the TCA cycle. Consistent with this result, we found that PC expression was enhanced in the 3D-spheroids (Supplementary Fig. S4C). Additionally, the total levels as well as ^13^C-enrichment of α-KG, glutamate, fumarate, malate, and aspartate were significantly higher in the 3D-spheroids (Fig. 2C). These results revealed robust glucose oxidation in the 3D-spheroids relative to 2D-cultured cells and demonstrated increased TCA cycle activity and capacity for ATP production via mitochondrial oxidative phosphorylation.

### Enhanced nucleotide synthesis and phosphorylation in 3D-spheroids

**Fig. 3:**
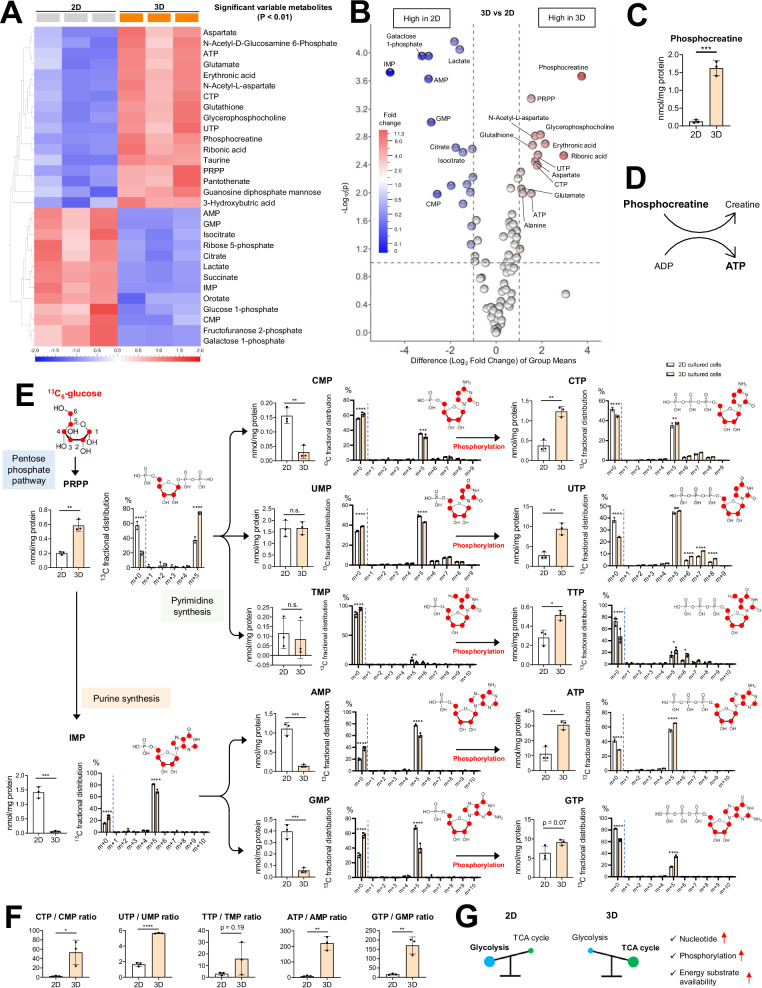
Enhanced nucleotide synthesis and phosphorylation in 3D-spheroids. **A** A heat map illustrating the comparison of metabolites between 2D- and 3D-cultured IGROV1 cells (Cutoff applied: *p*-value < 0.01). **B** A volcano plot revealing distinct metabolite signatures between 2D- and 3D-cultured IGROV1 cells. The volcano plot was generated to identify differentially enriched metabolites (Cutoff criteria: |Difference (Log_2_ Fold Change) of group means | > 1, and -Log_10_ (p-value) > 1). **C** Comparison of the levels of phosphocreatine in 2D- and 3D-cultured IGROV1 cells after 24 h incubation (*n* = 3 independent experiments). **D** Schematic illustrating the substrates and products of the creatine kinase reaction. **E** Comparison of intermediate levels for nucleotides and intermediates in 2D- and 3D-cultured IGROV1 cells after 24 h of incubation. The ^13^C isotopologue distribution of the metabolites is also illustrated. (*n* = 3 independent experiments). **F** Comparison of CTP/CMP, UTP/UMP, TTP/TMP, ATP/AMP, and GTP/GMP ratios in 2D- and 3D-cultured IGROV1 cells after 24 h incubation. (*n* = 3 independent experiments). **G** Schematic of 3D-spheroids activating the TCA cycle at higher levels than glycolysis, resulting in increased nucleotide production, phosphorylation, and energy substrate availability. The red circles indicate carbon. “m + 0” represents the carbon contribution from sources other than ^13^C_6_-glucose, and “m + 1,” “m + 2,” etc. represent the carbon contribution from ^13^C_6_-glucose. PRPP phosphoribosylpyrophosphate, IMP inosine monophosphate. Graph data were presented as mean ± SD of multiple experiments.

To comprehensively understand the metabolic differences between 2D- and 3D-cultured cells, we evaluated the levels of representative metabolites using a heat map and a volcano plot (Fig. 3A, B). Interestingly, the metabolite that exhibited the greatest increase in 3D-spheroids was phosphocreatine, whose main function was to serve as an energy buffer to maintain cellular ATP levels [29, 30] (Fig. 3C, D). Furthermore, 3D-spheroids contained higher levels of phosphoribosyl pyrophosphate (PRPP; Fig. 3E), a crucial component of nucleotide synthesis, which is derived from the pentose phosphate pathway (PPP) [31]. ^13^C-enrichment of PRPP was significantly greater in 3D-spheroids relative than in 2D-cultured cells, providing evidence of an enhanced capacity for nucleotide synthesis (Fig. 3E). Notably, the ^13^C-enrichment of nucleotides containing three phosphate groups, ATP, GTP, CTP, UTP, and TTP, was significantly higher in 3D-spheroids, while mono-phosphorylated nucleotides (IMP, AMP, GMP, CMP, UMP, and TMP) were less ^13^C-enriched in the 3D-spheroids (Fig. 3E). Both purines and pyrimidines exhibited robust m + 5 ^13^C-enrichment, indicating that their ribose moieties were derived from oxidative PPP. Pyrimidines, including UTP, also exhibited increased fractional enrichments of m + 6, m + 7, and m + 8 isotopologues (Fig. 3E), consistent with de novo pyrimidine synthesis from aspartate and orotate isotopologues partially labeled with ^13^C_6_-glucose (Fig. 2C and Supplementary Fig. S4D). We then examined the ratio of triphosphates/monophosphates to assess energy levels and observed that the CTP/CMP, UTP/UMP, ATP/AMP, and GTP/GMP ratios were notably higher, while the TTP/TMP ratio showed a tendency to increase (Fig. 3F). Overall, these results suggest that 3D-spheroids augmented their high-energy substrate levels by upregulating not only the synthesis, but also the phosphorylation of both purine and pyrimidine nucleotides, including ATP (Fig. 3G).

### NAMPT inhibitors are a potential strategy against NAMPT-dependent OV

**Fig. 4:**
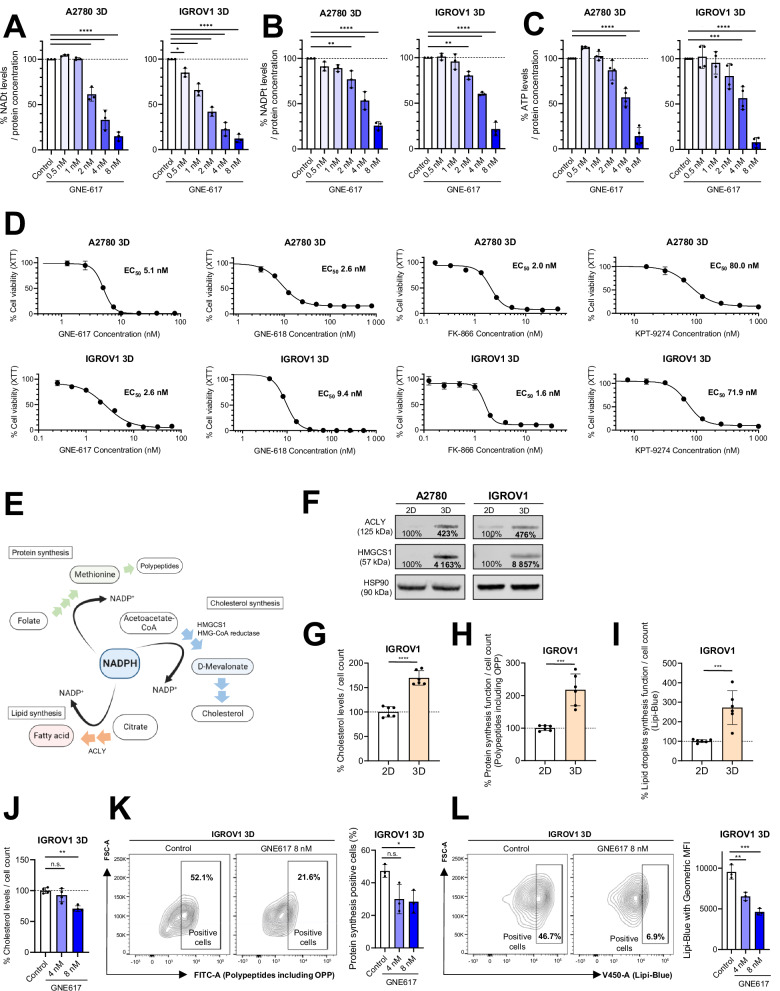
NAMPT inhibitors are a potential strategy against NAMPT-dependent OV. **A** Changes in total NAD levels in 3D-cultured A2780 and IGROV1 cells after treatment with GNE-617 for 24 h at the indicated doses (*n* = 3 independent experiments). **B** Changes in total NADP levels in 3D-cultured A2780 and IGROV1 cells after treatment with GNE-617 for 24 h at the indicated doses (*n* = 3 independent experiments). **C** Changes in total ATP levels in 3D-cultured A2780 and IGROV1 cells after treatment with GNE-617 for 24 h at the indicated doses (*n* = 4 independent experiments). **D** Relative cell viability after treatment with GNE-617, GNE-618, FK-866, and KPT-9274 for 72 h in 3D-cultured A2780 and IGROV1 cells (*n* = 4 independent experiments). **E** Schematic illustrating that NADPH is an essential co-enzyme for the synthesis of cholesterol, proteins, and fatty acids. HMGCS1: 3-hydroxy-3-methylglutaryl-CoA synthase 1, HMG-CoA: 3-hydroxy-3-methylglutaryl-co-enzyme A, ACLY: ATP citrate lyase. **F** Immunoblotting to evaluate ACLY and HMGCS1 expression in A2780 and IGROV1 cells, comparing 2D- and 3D-cultured cells. HSP90 is shown as a control. **G** Comparison of cholesterol concentrations normalized to the cell count in 2D- and 3D-cultured IGROV1 cells (*n* = 6 independent experiments). **H** Comparison of protein synthesis function normalized to the cell count using polypeptides including OPP in 2D- and 3D-cultured IGROV1 cells (*n* = 6 independent experiments). OPP: o-propargyl-puromycin. **I** Comparison of lipid droplet levels normalized to cell count using Lipi-Blue in 2D- and 3D-cultured IGROV1 cells (*n* = 6 independent experiments). **J** Changes in cholesterol concentration normalized to cell count in 3D-cultured IGROV1 cells after 48 h of treatment with GNE-617 at the indicated doses (*n* = 4 independent experiments). **K** Left: Representative figures illustrating the change in protein synthesis function using polypeptides including OPP (FITC), in 3D-cultured IGROV1 cells treated with GNE-617 at the indicated doses for 48 h. Cells within the rectangle represent polypeptides including OPP-positive cells. Right: Comparison of protein synthesis function using geometric MFI in 3D-cultured IGROV1 cells, as described above (*n* = 3 independent experiments). **L** Left: Representative figures illustrating the change in lipid droplet levels using Lipi-Blue (V450) in 3D-cultured IGROV1 cells treated with GNE-617 at the indicated doses for 48 h. Cells within the rectangle represent Lipi-Blue-positive cells. Right: Comparison of lipid droplet synthesis using geometric MFI in 3D-cultured IGROV1 cells, as described above (*n* = 3 independent experiments). Graph data were presented as mean ± SD of multiple experiments.

To further examine whether NAMPT may be an effective therapeutic target in OV, we first evaluated the effect of the NAMPT inhibitor GNE-617 on NAD^+^ production. We observed that GNE-617 effectively reduced NAD^+^ levels in 3D-cultured A2780 and IGROV1 cells, which are NAMPT dependent cell lines, in a concentration-dependent manner (Fig. 4A), similar to silencing NAMPT (Fig. 1F, G). GNE-617 significantly decreased intracellular NADPH (Fig. 4B) and ATP levels (Fig. 4C). In agreement with these results, GNE-617 and other NAMPT inhibitors, such as GNE-618, FK-866, and KPT-9274, significantly inhibited the growth of 3D-spheroids (Fig. 4D).

Given that NADPH is an essential co-enzyme for the synthesis of cholesterol, polypeptides, and fatty acids [32], we hypothesized that NAMPT inhibitors would also inhibit these processes (Fig. 4E). Protein levels of ATP citrate lyase (ACLY) and 3-hydroxy-3-methylglutaryl-CoA synthase (HMGCS1), which are essential enzymes for mevalonate and lipid synthesis, increased in 3D-cultured cells (Fig. 4F). Moreover, the cells had significantly higher cholesterol and polypeptide levels as measured by o-propargyl-puromycin (OPP), which reflects protein synthesis capacity, and increased Lipi-Blue staining, which reflects lipid-producing capacity (Fig. 4G–I). As anticipated, GNE-617 inhibited cholesterol synthesis in the 3D-spheroids (Fig. 4J). Similarly, the number and intensity of OPP-containing polypeptides and Lipi-Blue were significantly suppressed by GNE-617 treatment (Fig. 4K, L). Together, these results suggest that NAMPT inhibitors induce anti-tumor effects in NAMPT-dependent cancers by downregulating the production of NAD^+^, NADPH, and ATP, thereby affecting cholesterol, protein, and lipid synthesis.

### The combination of NAMPT inhibitors and disulfiram synergistically inhibits cell viability in vitro

**Fig. 5:**
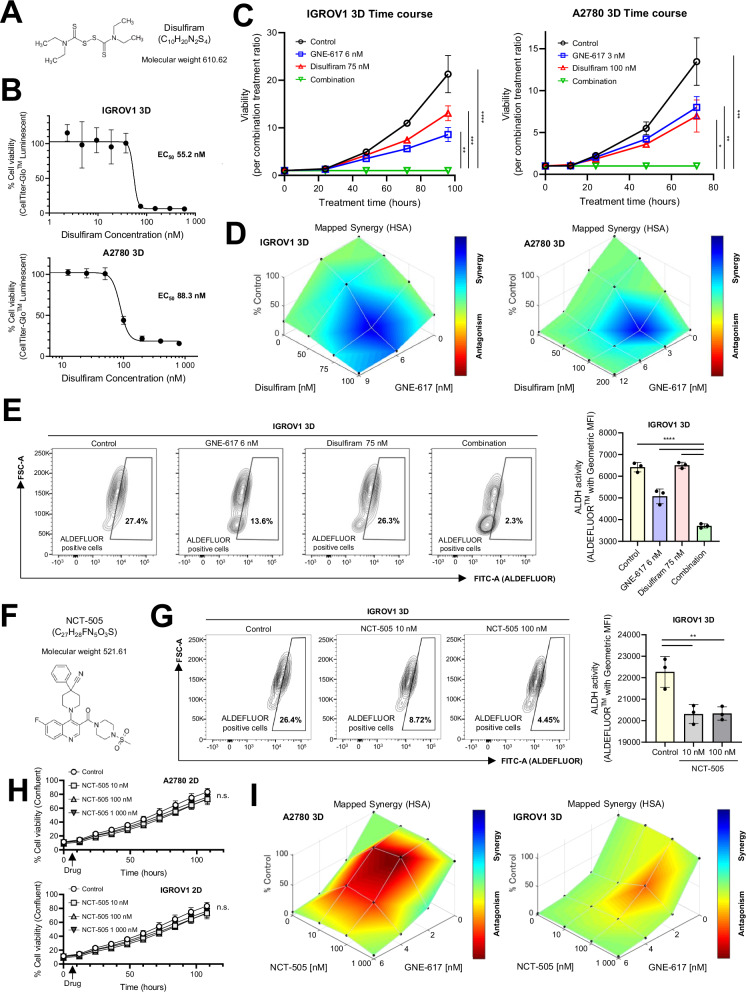
The combination of NAMPT inhibitors and disulfiram decreases the total number of viable cells. **A** Chemical structure of disulfiram. **B** Relative cell viability after treatment with disulfiram in 3D-cultured IGROV1 and A2780 cells for 72 h (*n* = 4 independent experiments). **C** The kinetic graph shows cell proliferation in 3D-cultured IGROV1 and A2780 cells with GNE-617, disulfiram, and combinatorial treatment at the indicated doses as a function of time (hours). The total number of viable cells with combinatorial treatment was set to 1 (*n* = 4 independent experiments). **D** Synergy plots generated by Combenefit showing the interaction between disulfiram and GNE-617 in 3D-cultured IGROV1 and A2780 cells. Analysis of the interaction resulted in HSA values (*n* = 4 independent experiments). **E** Left: Representative figures showing a change in ALDH activity using ALDEFLUOR fluorescence (FITC) in 3D-cultured IGROV1 cells after treatment with GNE-617, disulfiram, and combinatorial treatment at the indicated doses for 48 h. Cells included in the trapezoid represent ALDEFLUOR-positive cells. Right: Comparison of ALDH activity using geometric MFI in 3D-cultured IGROV1 as described above (*n* = 4 independent experiments). **F** Chemical structure of NCT-505. **G** Left: Representative images showing changes in ALDH activity using ALDEFLUOR fluorescence (FITC) in 3D-cultured IGROV1 cells after treatment with NCT-505 at the indicated doses for 48 h. Cells included in the trapezoid represent ALDEFLUOR-positive cells. Right: Comparison of ALDH activity using geometric MFI in 3D-cultured IGROV1 as described above (*n* = 3 independent experiments). **H** The kinetic graph shows cell proliferation in 2D-cultured IGROV1 and A2780 cells with NCT-505 at the indicated doses relative to Control over time (hours) (*n* = 4 independent experiments). **I** Synergy plots generated by Combenefit showing the interaction between NCT-505 and GNE-617 in 3D-cultured IGROV1 and A2780 cells. Analysis of the interaction resulted in HSA values (*n* = 4 independent experiments). Graph data were presented as mean ± SD of multiple experiments.

To enhance anti-tumor effects, delay drug resistance, and minimize side effects, we sought a drug that synergizes with NAMPT inhibitors. Previously, we showed that disulfiram (Fig. 5A), an ALDH inhibitor used to promote alcohol abstinence [33], inhibits ALDH activity, which is associated with stemness [34, 35]. ALDH consumes NAD^+^ to catalyze the oxidation of aldehyde substrates. Thus, we hypothesized that combining disulfiram with NAMPT inhibitors would yield synergistic outcomes. Disulfiram inhibited growth in 3D-spheroids alone (Fig. 5B), and in combination with GNE-617 demonstrated highly potent and synergistic anti-tumor effects in 3D-cultured IGROV1 and A2780 (Fig. 5C, D, and Supplementary Fig. S5A). Disulfiram also showed synergistic effects with other NAMPT inhibitors, including FK-866, GNE-618, and KPT-9274 (Supplementary Fig. S5A and S5B).

To evaluate the impact of combination treatment targeting ALDH activity, we measured ALDH activity using the ALDEFLUOR assay. We observed its effects on A2780 cells, characterized by low ALDH activity, and IGROV1 cells, which are known to exhibit high ALDH activity (Supplementary Fig. S5C). As anticipated, the combination treatment effectively suppressed ALDH activity in IGROV1 3D-spheroids (Fig. 5E). Notably, the combination therapy also exerted a synergistic anti-tumor effect in A2780 cells, despite their inherently low ALDH activity (Fig. 5C, D, and Supplementary Fig. S5A). These findings suggest that additional mechanisms, beyond the suppression of ALDH activity, may contribute to the synergistic effects observed in A2780 cells. To evaluate the synergistic effects of ALDH enzyme activity and understand the mechanism in detail, we also tested NCT-505, a specific inhibitor of ALDH1A1, which primarily contributes to ALDH activity [35], in combination with GNE-617 (Fig. 5F). Previous reports showed the ALDH inhibitory activity of NCT-505 at an IC_50_ of 2 nM in 3D-spheroids of the OV cell lines OVCAR3 and OV90 [35], and ALDH activity was significantly suppressed at 10 and 100 nM of NCT-505 in 3D-cultured IGROV1 cells (Fig. 5G). Despite strong suppression of ALDH activity, NCT-505 showed no obvious anti-proliferative effect on itself in IGROV1 (Fig. 5H). Similarly, no anti-tumor effect of NCT-505 was shown in A2780 (Fig. 5H). When NCT-505 was combined with GNE-617, an antagonistic effect, rather than a synergistic effect, was observed in both IGROV1 (high ALDH activity-high) and A2780 (low ALDH activity) (Fig. 5I). Our findings not only suggest that the combination of disulfiram and NAMPT inhibitors could be an effective strategy against OV but also indicate an unknown ALDH activity-independent mechanism that drives the synergistic effects of disulfiram and NAMPT inhibitors.

### GNE-617 inhibits the GAPDH-mediated response and PAR production, while disulfiram suppresses the TCA cycle

**Fig. 6:**
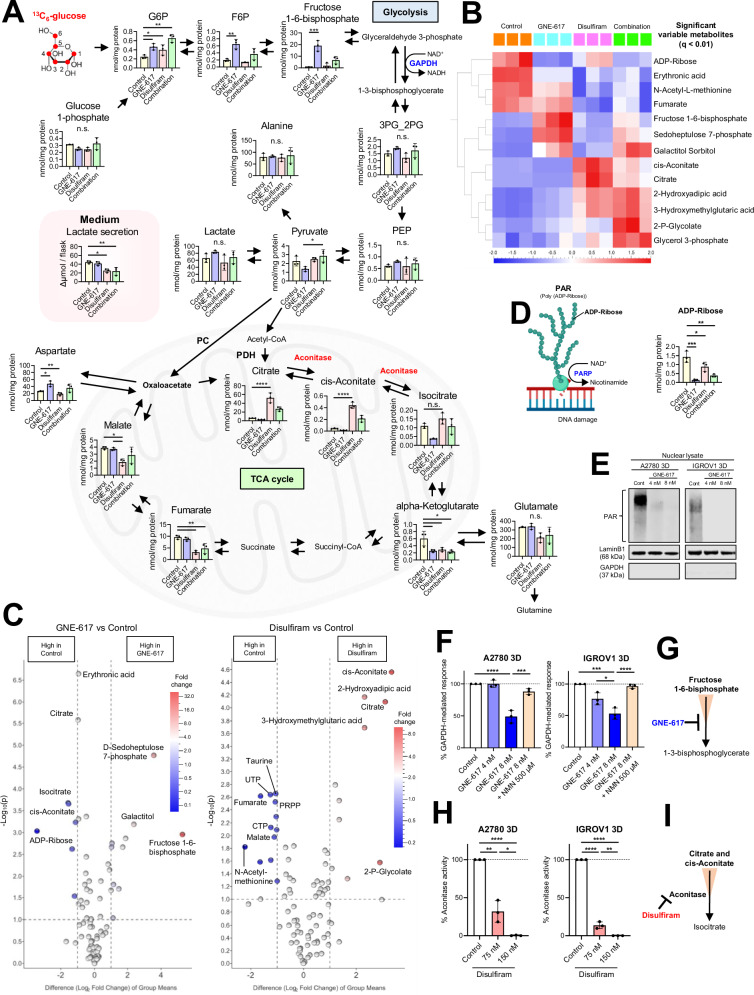
GNE-617 inhibits the GAPDH-mediated response and PAR production, while disulfiram suppresses the TCA cycle in 3D-cultured OV. **A** Schematic showing the key metabolites of glycolysis and TCA cycle in 3D-cultured IGROV1 cells after treatment with GNE-617, disulfiram, and combinatorial treatment at indicated doses for 24 h. Red circles indicate carbon. Additionally, the secretion of lactate into the medium after 24 h of incubation is presented (*n* = 3 independent experiments). G6P glucose-6-phosphate, F6P fructose-6-phosphate, 3PG_2PG, 3-phosphoglycerate, 2-phosphoglycerate, PEP phosphoenolpyruvate, α-KG alpha-ketoglutarate, PC pyruvate carboxylase, PDH pyruvate dehydrogenase. **B** A heat map illustrating the comparison of metabolites in 3D-cultured IGROV1 cells as described above (cutoff applied: q-value < 0.01). **C** Left: Volcano plot revealing distinct metabolite signatures after treatment with GNE-617 in 3D-cultured IGROV1 cells. A volcano plot was generated to identify differentially enriched metabolites (cutoff criteria: | difference (Log_2_ Fold Change) of group means | > 1 and -Log_10_ (p-value) > 1). Right: Volcano plot revealing distinct metabolite signatures after treatment with disulfiram in 3D-cultured IGROV1 cells. A volcano plot was generated to identify differentially enriched metabolites (cutoff criteria: | difference (Log_2_ Fold Change) of group means | > 1 and -Log_10_ (*p*-value) > 1). **D** Left: Schematic showing that poly(ADP-ribos)ylation (PARylation), a repair process for damaged DNA, is mediated by NAD^+^ as a co-factor. Right: Change in the levels of ADP-ribose in 3D-cultured IGROV1 cells, as described above (*n* = 3 independent experiments). **E** Immunoblotting for evaluating PAR expression in 3D-cultured A2780 and IGROV1 nuclear cell lysates after treatment with GNE-617 at the indicated doses for 48 h. LaminB1 and GAPDH were used as controls. **F** Changes in GAPDH-mediated response normalized to the protein concentration in 3D-cultured IGROV1 after treatment with GNE-617 at the indicated doses for 48 h. NMN was added to the media at the indicated doses to confirm NMN rescue (*n* = 3 independent experiments). **G** Schematic showing that GNE-617 treatment leads to the build-up of fructose 1-6-bisphosphate. **H** Changes in aconitase activity normalized to the protein concentration in 3D-cultured IGROV1 cells after treatment with disulfiram at the indicated doses for 48 h (*n* = 3 independent experiments). **I** Schematic showing inhibition of aconitase resulting from disulfiram treatment. Graph data were presented as mean ± SD of multiple experiments.

To elucidate the mechanisms that induce the synergistic effect of combinatorial treatment, we tested metabolite changes induced by GNE-617, disulfiram, and combinatorial treatment in 3D-spheroids grown in the presence of ^13^C_6_-glucose for 24 h during treatments (Supplementary Fig. S6A). Principal component analysis revealed significant differences between the four treatment groups (Supplementary Fig. S6B). We evaluated the levels of representative metabolites of glycolysis and the TCA cycle (Fig. 6A). To identify differentially expressed metabolites, we compared the effects of each treatment (Fig. 6B, C). GNE-617 markedly downregulated ADP-ribose produced from NAD^+^ in the mitochondria (Fig. 6B–D). Given that poly(ADP-ribos)ylation (PARylation), a repair process for damaged DNA, is mediated by NAD^+^ as a co-factor [36] (Fig. 6D), we hypothesized that GNE-617 would also inhibit PARylation. As anticipated, GNE-617 treatment markedly suppressed PAR protein expression (Fig. 6E). Fructose 1-6-bisphosphate levels increased 10-fold following GNE-617 treatment (Fig. 6A). To test our hypothesis that GNE-617 inhibits the flux from glyceraldehyde-3-phosphate to 1-3-bisphosphoglycerate via GAPDH, which results in the accumulation of fructose 1-6-bisphosphate, we quantified the GAPDH-mediated response. GNE-617 downregulated GAPDH-mediated flux in a concentration-dependent manner, and this effect was rescued by NMN (Fig. 6F). This suggested that GNE-617 blocked the conversion of fructose 1-6-bisphosphate to glyceraldehyde-3-phosphate (Fig. 6G). Compared with the control, disulfiram significantly reduced the metabolite levels of α-KG, fumarate, malate, and aspartate (Fig. 6A), as well as a notable decrease in glucose contribution to these metabolites (Supplementary Fig. S6C), indicating decreased capacity for glucose oxidation via the TCA cycle. In further support of this, we found that the levels of citrate and cis-aconitate in the disulfiram-treated group were more than 20 times higher than in the control group (Fig. 6A). To test the hypothesis that citrate and cis-aconitate accumulation is caused by the inhibition of aconitase, an enzyme that catalyzes the isomerization of citrate to isocitrate via cis-aconitate [27], we directly measured aconitase in disulfiram-treated cells. Disulfiram treatment significantly reduced the aconitase activity in both A2780 and IGROV1 cells (Fig. 6H, I). Moreover, disulfiram, alone or in combination, significantly diminished the glucose contribution to m + 3 and m + 4 isotopologues of citrate, whereas the m + 2 citrate isotopologue increased, consistent with a block of the TCA cycle at the level of aconitase [27] (Supplementary Fig. S6C). Taken together, these findings suggest that GNE-617 inhibits ADP-ribose production derived from the mitochondria, while disulfiram acts as an aconitase inhibitor within the TCA cycle. This indicates an anti-tumor mechanism involving the suppression of mitochondrial function.

### Combinatorial treatment induces a synergistic anti-tumor effect by suppressing mitochondrial function and triggers cell cycle arrest in vitro

**Fig. 7:**
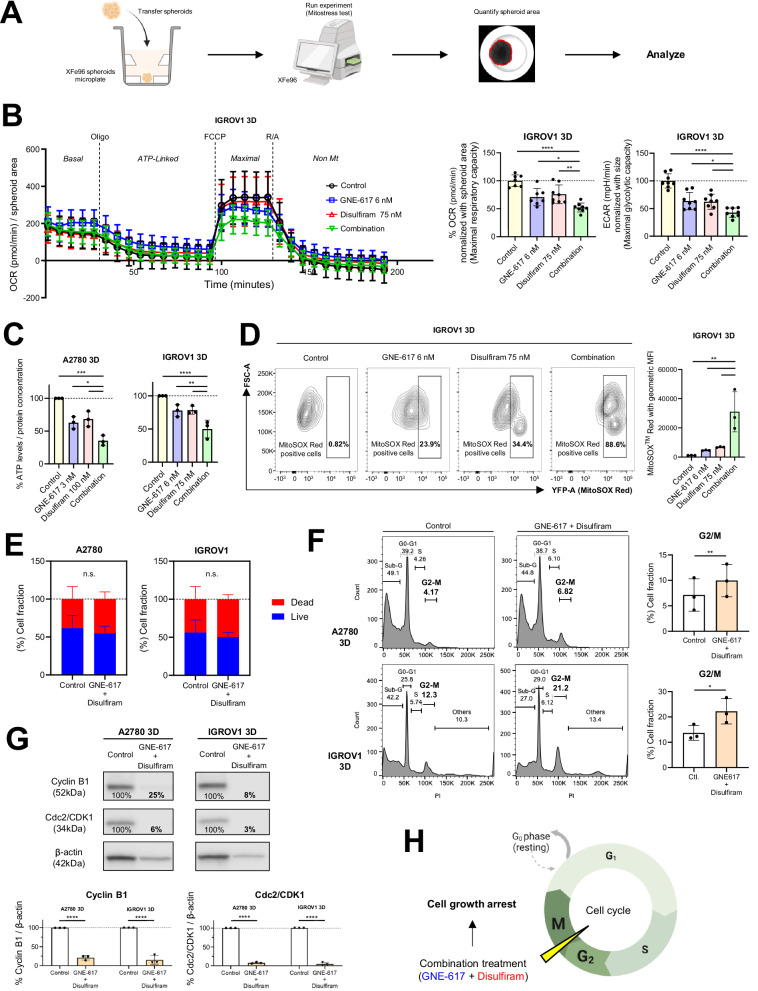
Combinatorial treatment induces a synergistic anti-tumor effect by suppressing mitochondrial function and triggers cell cycle arrest in 3D-cultured OV. **A** Schematic illustrating the process of the Mito stress test using 3D-spheroids. **B** Left: Representative OCR patterns in 3D-cultured IGROV1 spheroids after treatment with GNE-617, disulfiram, and combinatorial therapy at the indicated doses for 48 h over time (min), normalized to spheroid area. Oligomycin, FCCP, rotenone, and antimycin A were added to measure Basal OCR, ATP-Linked OCR, maximal OCR, and non-mitochondrial OCR (*n* = 8 technical replicates). Middle: Maximal respiratory capacity in OCR (*n* = 8 technical replicates). Right: Maximal glycolytic capacity in ECAR (*n* = 8 technical replicates). **C** Changes in total ATP levels normalized to protein concentration in 3D-cultured A2780 and IGROV1 cells using the same cells as in (**B**) (*n* = 3 independent experiments). **D** Left: Representative figures showing changes in ROS production from mitochondria using MitoSOX Red (YFP) in the same cells as in (**B**). Cells within the rectangle represent MitoSOX Red-positive cells. Right: Comparison of MitoSOX Red using geometric MFI in 3D-cultured IGROV1 cells as described above (*n* = 3 independent experiments). **E** Relative fractions of live and dead cells in 3D-cultured A2780 and IGROV1 cells were analyzed using the AOPI assay after 96 h of treatment with either a DMSO control or the combination of GNE-617 (30 nM) and disulfiram (600 nM) (*n* = 6 independent experiments). **F** Left: Representative images of cell cycle analysis performed using Propidium Iodide (PI) staining and flow cytometry. 3D-spheroids were treated with either a DMSO control or a combination of GNE-617 and disulfiram for 96 h. Right: Quantitative comparison of the G2/M phase fraction between the control and combination drug treatment groups (*n* = 3 independent experiments, paired *t*-test). **G** Top: Immunoblot analysis of Cyclin B1 and Cdc2/CDK1 protein levels in 3D-cultured A2780 and IGROV1 cells treated with either DMSO control or the combination of GNE-617 and disulfiram. β-actin was used as a loading control. Bottom: Comparison of normalized protein levels relative to β-actin (*n* = 3 independent experiments). **H** Schematic representation illustrating how the combination treatment induces cell growth arrest by inhibiting the G2 to M phase transition. Graph data were presented as mean ± SD of multiple experiments.

To evaluate whether the combinatorial treatment downregulated mitochondrial function, we first quantified the anti-tumor effect of GNE-617 and disulfiram on mitochondrial respiration in 3D-spheroids (Fig. 7A). We observed that the combination treatment markedly inhibited both maximal respiratory capacity and spare respiratory capacity (Fig. 7B and Supplementary Fig. S7A). Furthermore, the treatment significantly suppressed not only OCR but also maximal ECAR, which reflects glycolytic activity (Fig. 7B). Additionally, we found reduced expression of Complex-V, an ATP synthase in the mitochondria [37] (Supplementary Fig. S7B). Consistent with the reduction in OCR and Complex-V, we observed that the combinatorial treatment inhibited ATP production in 3D-spheroids (Fig. 7C). We next used MitoSOX Red to quantify mitochondrial-derived reactive oxygen species (ROS), which reflects the presence of damaged mitochondria [38]. The expression of MitoSOX Red was enhanced by the combinatorial treatment compared to monotherapy (Fig. 7D and Supplementary Fig. S7C and S7D).

Next, we examined the effect of combination therapy on cell viability and cytotoxicity in 3D-spheroids. While the combination therapy significantly suppressed Realtime MT Glo signals, which indicates the total number of viable cells, it did not increase CellTox Green fluorescence, a marker of cell death (Supplementary Fig. S7E) even with higher concentrations of drugs (GNE-617 30 nM and disulfiram 600 nM). Additionally, the pan-caspase inhibitor benzyloxycarbonyl-Val-Ala-Asp-fluoromethylketone (Z-VAD-FMK), the potent blood-brain barrier-penetrant necroptosis inhibitor necrostatin-1 (Nec1), and the selective ferroptosis inhibitor ferrostatin had no impact on the total number of viable cells and cytotoxicity (Supplementary Fig. S7E). Furthermore, the combination therapy did not alter the live-to-dead cell ratio in the AOPI assay (Fig. 7E). These findings suggest that the anti-tumor effects of the combination therapy are mediated through mechanisms independent of cell death induction.

As a next step, we assessed cell cycle distribution by flow cytometry and observed that the combination therapy significantly increased the G2/M phase fraction (Fig. 7F). Furthermore, the treatment markedly downregulated the expression of Cyclin B1 and Cdc2/CDK1 (Fig. 7G), key factors required for M phase initiation [39, 40]. Collectively, these findings suggest that the combination therapy inhibited the cell cycle, particularly the G2 to M phase transition, and suppressed cell proliferation through mitochondrial dysfunction (Fig. 7H).

### Combinatorial treatment significantly inhibits tumor growth and extends survival in vivo

**Fig. 8:**
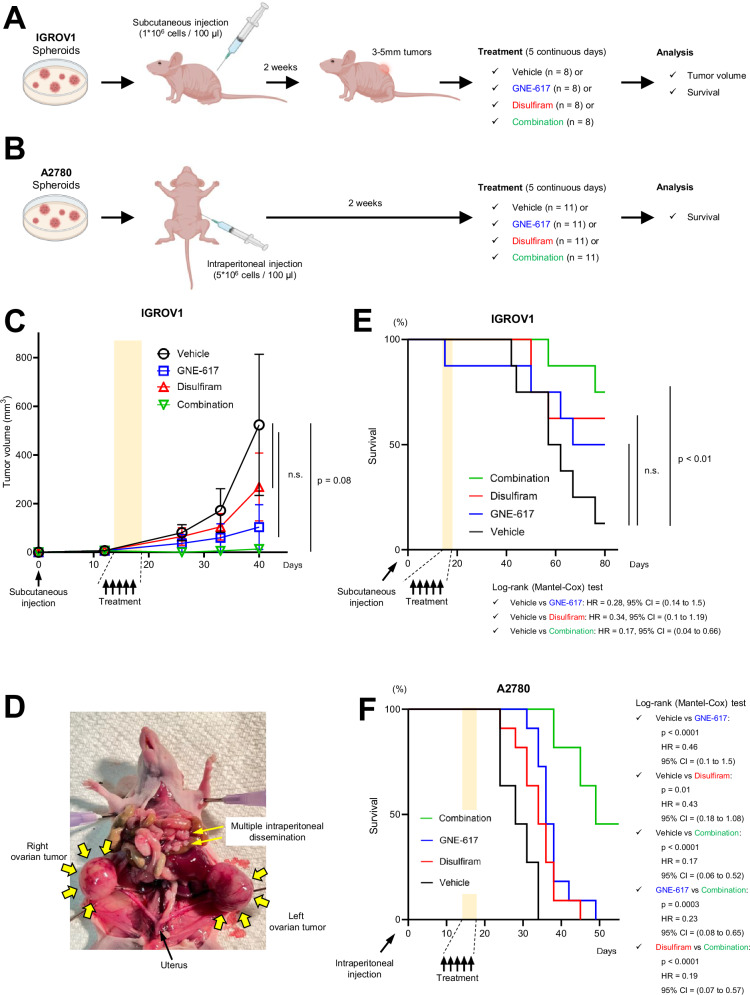
Combinatorial treatment significantly inhibits tumor growth and extends survival in vivo. **A** Schematic illustrating animal experiments in which 3D-cultured IGROV1 cells were subcutaneously injected into mice, followed 2 weeks later with vehicle control, GNE-617, disulfiram, or the combinatorial treatment for 5 consecutive days. **B** Schematic illustrating animal experiments in which 3D-cultured A2780 cells were intraperitoneally injected into mice, followed 2 weeks later with the same treatment as in (**A**). **C** The kinetic graph shows depicting tumor volume over time (days) with the same treatment as in (**A**) (*n* = 8). **D** Representative image showing remarkable intraperitoneal dissemination and bilateral ovarian enlargement approximately 4 weeks after intraperitoneal injection of tumor cells. **E** Survival curves for mice injected subcutaneously with IGROV1 cells (*n* = 8). **F** Survival curves for mice injected intraperitoneally with A2780 cells (*n* = 11). Graph data were presented as mean ± SD of multiple experiments.

We tested the effects of the combinatorial therapy in two immunocompromised mouse models. In the first experiment, IGROV1 spheroids were subcutaneously implanted, and in the second experiment, A2780 spheroids were intraperitoneally injected into immunocompromised mice (Fig. 8A, B). In both experiments, the mice were randomly divided into four groups: vehicle control, GNE-617, disulfiram, or the combinatorial treatment for five continuous days (Fig. 8A, B). In the subcutaneous model, beginning on day 40, mice in the control and subsequently in other groups had to be euthanized due to their tumors exceeding the upper limit described in the NIH Animal Care and Use Committee protocol. Therefore, size comparisons were stopped after day 40. We observed a trend for decreased tumor growth in subcutaneous tumors with combinatorial treatment (Fig. 8C). Within the group receiving combinatorial treatment in the intraperitoneal model, two mice exhibited a transient reduction in body weight post-therapy, which subsequently stabilized (Supplementary Fig. S8C, S8D). In the intraperitoneal model, all untreated mice exhibited intraperitoneal dissemination and ovarian enlargement (Fig. 8D), leading to euthanasia 3-5 weeks after injection. Notably, the combinatorial treatment significantly increased mouse survival (Fig. 8E, F). Taken together, our findings indicate that the synergistic effect of GNE-617 and disulfiram reduces tumor growth and enhances survival in OV-bearing mice. This suggests that such a combinatorial approach may offer a novel and potent therapeutic strategy for the management of recurrent OV.

## Discussion

CSCs contribute to OV recurrence and metastasis due to their resistance to therapy and their ability to repopulate tissues [8]. In our previous work, there are several reports that spheroids derived from OV cell lines and primary OV tissue contain abundant CSC-like cells that exhibit high ALDH activity, increased expression of stem cell markers, self-renewal, high proliferation and differentiation potential [10, 41]. In this study, we demonstrated that 3D-spheroids morphologically and functionally mimic CSCs enriched in ascites, exhibiting unique cancer metabolism. Several other spheroid production methods have been reported, including liquid overlay [42], hanging drop [43], spinner culture [44], rotating wall vessel [44], microfluidics [44], and magnetic levitation [44]. The culture method used in this study, using ULA plates with hydrophilic, biologically inert, non-degradable surface coatings, enables the rapid and highly reproducible formation and growth of single spheroids per well [44]. Therefore, 3D-spheroids with ULA plates are valuable preclinical models for exploring the mechanisms of recurrence and metastasis.

Metabolomic analyses utilizing (NMR and IC-MS) showed elevated total levels as well as ^13^C-enrichment of TCA cycle-related metabolites (α-KG, glutamate, fumarate, malate, and aspartate) in 3D-spheroids, consistent with prior the results [45]. The most parsimonious explanation for the presence of m + 2 isotopologues in TCA cycle intermediates is the condensation of ^13^C_6_-glucose-derived acetyl-CoA, arising from the activity of pyruvate dehydrogenase, with unlabeled oxaloacetate via the citrate synthase reaction [27, 28]. Similarly, m + 4 TCA cycle intermediates can arise from the condensation of m + 2 acetyl-CoA with m + 2 oxaloacetate derived from the full turn of the TCA cycle [27, 28]. Finally, m + 3 and m + 5 isotopologues of citrate/isocitrate, α-KG, and glutamate can arise from the activity of PC and support anapleurotic TCA cycle activity [27, 28]. Notably, we observed a citrate ^13^C isotopologue distribution pattern in 3D-spheroid cultures consistent with both enhanced glucose oxidation and increased capacity for anapleurosis. The decreased total amount of citrate, as well as lower fractional enrichment of the m + 2 citrate isotopolog in our 3D-spheroids, is consistent with the diversion of citrate into the cytosol, where it can be cleaved by ACLY to form cytosolic acetyl-CoA, which can be utilized for lipid and cholesterol synthesis [46, 47]. Indeed, our observation of increased cholesterol and lipid droplet levels in 3D-spheroids is consistent with cytosolic citrate cleavage. Finally, nucleotide synthesis and phosphorylation are activated in 3D-spheroids, implying a high-energy state linked to therapeutic resistance and malignancy. These findings uncover the mechanisms underlying OV dissemination and recurrence and offer potential directions for novel therapies. However, as glucose is not the sole source of carbon atoms in the TCA cycle, further studies using other stable isotope tracers, such as ^13^C_5_-glutamine, are warranted to comprehensively understand spheroid-specific metabolism.

We also showed that high NAMPT expression was a poor prognostic indicator among several rate-limiting enzymes in NAD^+^ production pathways and a potential new therapeutic target in OV. Analysis of publicly available datasets suggested a strong correlation between high NAMPT expression and adverse patient outcomes in OV patients. Interestingly, according to a previous study [48], NAMPT inhibition was reported to be cytotoxic in cancer cell lines in vitro but not in non-cancer cells. Additionally, in vivo experiments have confirmed its anti-tumor efficacy against OV, offering hope for clinical applications [49, 50]. Nevertheless, identifying predictive biomarkers and therapeutic strategies for spheroids that generate NAD^+^ independently of NAMPT, utilizing NA and Trp, remains a challenge and necessitates further studies. We also found that the combination of NAMPT inhibitors and disulfiram exerted a robust synergistic anti-tumor effect that effectively inhibited cell proliferation in vitro and extended survival in vivo. We emphasize that the use of NAMPT inhibitors in combination with disulfiram, as demonstrated in this study, should be restricted to OV cases with confirmed NAMPT dependence. This strategy represents a molecularly targeted therapy specifically tailored for NAMPT-dependent OV. In these animal studies, only one course of treatment (five continuous days of administration) was conducted to evaluate the effect of the combined treatment, but there is a possibility that further anti-tumor effects and prolongation of survival could be expected by increasing the number of treatment courses and the duration of maintenance therapy. Although there is still room for further investigation of the side effects associated with combinatorial treatment, such as weight loss, this approach is expected to be a promising strategy for recurrent OV.

The observation that NCT-505, an ALDH1A1-specific inhibitor, did not synergize with GNE-617 suggested the existence of an alternative mechanism for inducing synergistic effects beyond ALDH1 activity inhibition. Metabolomics data analysis, conducted to elucidate the mechanism in detail, revealed that the combinatorial treatment significantly affected central carbon metabolism and mitochondrial function. Interestingly, this analysis also revealed that disulfiram acts as an aconitase inhibitor, which has not been previously reported. This finding is novel and elucidates a part of the mechanism underlying the anti-tumor effect of disulfiram. However, several unanswered questions remain, such as the mechanism by which disulfiram inhibits aconitase activity (directly or indirectly), and whether aconitase activity is also inhibited in patients undergoing clinical disulfiram treatment. Therefore, further studies are warranted.

Furthermore, we found that the anti-tumor effect of the combination therapy is primarily mediated by inhibiting the cell cycle, particularly the transition from the G2 to M phase, rather than by actively inducing cell death. Considering that energy demand peaks during mitosis [51], ATP deficiency caused by the combination therapy likely prevents cells from entering mitosis, thereby suppressing cell proliferation. Additionally, the activity of sirtuins, which regulate the cell cycle and DNA repair, depends on NAD^+^ [52], and the suppression of NAD^+^ production through NAMPT inhibition may have contributed to the arrest of the G2 to M phase transition in 3D-spheroids. On the other hand, oxidative stress is also a significant factor associated with G2/M arrest [53]. The increase in ROS induced by the combination therapy may have further contributed to cell cycle inhibition.

In conclusion, our study underscores the potential of spheroids as a valuable preclinical model for OV, and highlights NAMPT inhibition as a novel therapeutic approach for OV. Moreover, in vitro and in vivo model studies have indicated that combining NAMPT inhibitors and disulfiram is a novel and effective strategy to overcome recurrent OV.

## Materials And Methods

### Antibodies and chemicals

The following primary antibodies were purchased from Cell Signaling Technology (MA, USA) and used at the indicated dilutions for western blot analysis: CD44 (#3570S, 1:1000), CD133 (#64326S, 1:1000), PBEF/NAMPT (#86634S, 1:1000), lamin B1 (#12586S, 1:1 000), hexokinase II (#2867S, 1:1000), ATP-Citrate Lyase (#13390S, 1:1000), HMGCS1 (#36877S, 1:1000), and Cdc2/CDK1 (#77055, 1:1000). The following primary antibodies were purchased from Abcam (Cambridge, UK): NAPRT1 (#ab211529, 1:1000), QPRT (#ab171944, 1:1000), and NADSYN1 (#ab171942, 1:1000). HSP90α/β (#sc-13119, 1:1000) antibody and beta-actin HRP (#sc-47778 HRP) were obtained from Santa Cruz Biotechnology (TX, USA). GAPDH (#60004-1-Ig, 1:1000) antibody was purchased from Proteintech (IL, USA). Poly (ADP-ribose) (#10407, 1:2000) antibody was obtained from Immuno-Biological Laboratories (Gunma, Japan). Pyruvate Carboxylase (#PA5-50101, 1:1000) and OxPhos Human WB Antibody Cocktail (#45-8199, 1:1000) were purchased from Thermo Fisher Scientific (MA, USA). Cyclin B1 antibody (#554179, 1:1000) was purchased from BD Pharmingen (NJ, USA). The following compounds were purchased from the indicated suppliers for in vitro studies: FK-866 (#HY-50876, MedChemExpress, NJ, USA), GNE-617 (#HY-15766, MedChemExpress), GNE-618 (#HY-12628, MedChemExpress), disulfiram (#PHR1690-1G, Millipore Sigma), β-nicotinamide mononucleotide (NMN) (#N3501-25MG, Millipore Sigma), and nicotinic acid (NA) (#N4126-100G, Millipore Sigma). The PAK4-NAMPT dual inhibitor (KPT-9274) was kindly provided by Karyopharm Therapeutics (Newton, MA, USA). The ALDH1A1 inhibitor, NCT-505, was provided by Dr. Shyh-Ming Yang (National Center for Advancing Translational Sciences, MD, USA). Cell death inhibitors, including Z-VAD-FMK (#S7023), Necrostatin-1 (#S8037), ferrostatin-1 (#S7243), were purchased from Selleckchem (TX, USA).

### Cell lines and tissue culture

OV cell lines (A2780, IGROV1, OVCAR8, OVCAR8, ES2, and SKOV3) were purchased from American Type Culture Collection (ATCC, VA, USA). Cells were cultured at 37°C in a 5% CO_2_ environment in RPMI-1640 (#11875093, Thermo Fisher Scientific) medium supplemented with 10% fetal calf serum (FCS) (#100-106, GeminiBio, CA, USA) and 1% penicillin (100 units/ml)/streptomycin (100 units/ml) (#15140-122, Thermo Fisher Scientific). For 3D-spheroids, ultra-low attachment plates (ULA) (#3471, 3474, 3814, and 7007, Corning, NY, USA) were used with Stem Cell culture media consisting of 1% KnockOut serum replacement (#10828-010, Thermo Fisher Scientific), 1% penicillin/streptomycin, 0.1% insulin-transferrin-selenium (#41400-045, Thermo Fisher Scientific) and 0.4% bovine serum albumin (#A9418, Millipore Sigma). Mycoplasma infection was prevented using prophylactic Plasmocin (#ant-mpp, InvivoGen, CA, USA).

### Western blot

Cells were rinsed with PBS and lysed using 0.5% NP-40 (#13021, Millipore Sigma) with a Halt protease and phosphatase inhibitor cocktail (#78442, Thermo Fisher Scientific). Cytoplasmic and nuclear lysates were prepared using a Rapid, Efficient And Practical (REAP) method [54]. The cell pellets were resuspended in ice-cold 0.5% NP-40 in PBS and centrifuged at 10,000 g for 10 s at 4 °C. The supernatant was removed and used as the cytoplasmic lysate. The pellet was resuspended in 1 ml of ice-cold 0.5% NP-40 in PBS and centrifuged as described above for 10 s, and the supernatant was discarded. The pellet was resuspended in 0.5% NP-40 in PBS and designated as the nuclear lysate. The bicinchoninic acid (BCA) method (#23227, Thermo Fisher Scientific) was used for protein quantification. Lysates were boiled for 5 min, resolved using NuPAGE 4-12% SDS-PAGE gels (#NP0335BOX, Thermo Fisher Scientific), and transferred to nitrocellulose membranes (#IB23002, Thermo Fisher Scientific) using an iBlot2 Dry Blotting System (#IB21001, Thermo Fisher Scientific), or Immobilon-P PVDF membrane (#IPVH00010, Millipore-Sigma) using Criterion blotter system (Bio-Rad). The membranes were blocked using Intercept Blocking Buffer (#927-60001, LI-COR Biosciences), probed with primary antibodies overnight at 4 °C, and probed with secondary antibodies at room temperature for 1 h. Immune complexes were visualized with either fluorescence- or chemiluminescence-based detection system using Odyssey Fc Imager (LICOR BioSciences, NE, USA). The fluorescence detection, IRDye 800CW (#926-32210 anti-mouse and #926-32211 anti-rabbit, 1:5000) and IRDye 680RD (#926-68070 anti-mouse and #926-68071 anti-rabbit, 1:5000, LI-COR Biosciences) were used. For chemiluminescence detection, Goat Anti-Rabbit IgG (H + L)-HRP Conjugate and Goat Anti-Mouse IgG (H + L)-HRP Conjugate (#172-1019, #172-1011, 1:2000, Bio-Rad Laboratories, Hercules, CA, USA), and SuperSignal West Pico PLUS and Femto substrate (#34580 and #34095, Thermo Fisher) were used.

### Reverse transcriptase-quantitative PCR

Cells cultured in 2D or 3D conditions for 96 h were collected, and total RNA was isolated with RNeasy mini kit (#74104, Qiagen, Venlo, Netherlands). Reverse transcriptase reaction was performed with QuantiTect Reverse Transcription Kit (#205311, Qiagen). PCR reaction was performed with 2x PowerUp SYBER Green master mix (#A25777, Thermo Fisher Scientific) and ViiA 7 Real-Time PCR system (Thermo Fisher). Primers used for qPCR are listed in the table. All qPCR was performed with three technical replicates with multiple biological replicates. PCR cycle condition was 50 °C (2 min), 95 °C (2 min), followed by 40 cycles of two-step cycling of 95 °C (15 s) and 60 °C (60 s). Ct value of each target gene was obtained from qPCR results. Results were quantitatively analyzed with ΔΔCt method. First, average ΔCt (Ct [target gene] − Ct [ACTB]) in 2D samples were obtained, then each ΔΔCt was obtained by (ΔCt - average ΔCt). Lastly, 2^-ΔΔCt^ was used as relative copy number of each transcript.

### NAD^+^, NADPH, and ATP concentration measurements

NAD^+^ levels were assessed using the NAD^+^/NADH Quantification Colorimetric Kit (#K337-100, Biovision), NADPH levels were assessed using the NADPH Quantitation Fluorometric Assay Kit (#K349-100, Biovision), and ATP levels were assessed using the ATP Colorimetric/Fluorometric Assay Kit (#K354-100, Biovision), according to the manufacturer’s instructions. The results were normalized by protein concentration and measured using a SpectraMax i3 microplate spectrophotometer (Molecular Devices, CA, USA).

### MitoTracker Orange CMTMRos and MitoTracker Green FM fluorescence measurements

The activity and localization of mitochondria in living cells were evaluated using MitoTracker Orange CMTMRos (#M7510, Thermo Fisher Scientific) and MitoTracker Green FM (#M7514, Thermo Fisher Scientific) according to the manufacturer’s instructions. The number of trypsinized cells on 96-well plates was obtained by counting Hoechst-stained cells. The results were normalized to cell counts and measured using SpectraMax i3.

### Cell viability and cytotoxicity analysis

Cells were cultured in 96-well plates at various densities, depending on the growth rate. Cell viability was assessed using 4 different assays. The XTT assay (#11465015001, Millipore Sigma) measures the total number of viable cells based on the reduction of yellow tetrazolium salt (XTT) into an orange formazan dye. The CellTiter-Glo assay (#G7570, Promega, WI, USA) is a homogeneous method that quantifies viable cells by measuring ATP levels, while the RealTime-Glo MT assay (#G9711, Promega) determines the total number of viable cells through an ATP-independent mechanism. MTS cell viability assay (Cell Titer 96 AQueous One Solution Cell Proliferation Assay, #G3581, Promega) was also used to assess the total number of viable cells. To measure the cytotoxicity, CellTox Green assay (#G8741, Promega) was used. Data were normalized to the median value of control wells and analyzed using the SpectraMax i3 system.

### Acridine Orange/Propidium Iodide (AOPI) cell viability assay

Live/dead and total cell numbers were counted with Cellometer ViaStain AOPI Staining Solution (#CS2-0106-5 mL, Nexcelom Bioscience, CA, USA) using Cellometer K2 Fluorescent Cell Counter.

### Stable Isotope-Resolved Metabolomics (SIRM) workflow

Cells were incubated in DMEM/F12 (#PM150322, Elabscience, TX, USA) containing 10 mM unlabeled glucose (#A2494001, Thermo Fisher Scientific), 10% fetal calf serum (FCS) (#100-106, GeminiBio, CA, USA), 1% penicillin/streptomycin, and 0.1% insulin-transferrin-selenium. Six days after cell seeding, the culture medium was replaced with tracer medium consisting of DMEM/F12 supplemented with 10 mM [U-^13^C]-glucose (#CLM-1396-10, Cambridge Isotope Laboratories, MA, USA) and 10% dialyzed FBS containing vehicle, GNE-617, disulfiram, or both. After 24 h of growth, the cells were washed twice with ice-cold PBS and quenched with 100% acetonitrile at −20 °C. Metabolites were extracted using the Fan Extraction Method [55] with lyophilized aqueous fractions for nuclear magnetic resonance (NMR) and mass spectrometry, while the organic fraction was stored at −80 °C in chloroform in the presence of butylated hydroxytoluene (BHT), and the protein fraction was dried in a vacuum centrifuge. Glucose and lactate concentrations in the culture media were measured using a YSI 2950 Bioanalyzer (#527690, YSI, OH, USA).

### NMR Analysis

A lyophilized polar metabolite fraction was dissolved in D_2_O solution containing 0.25 μM DSS-d6 as reference and concentration standard. 1D ^1^H Presat and ^1^H-^13^C heteronuclear single quantum coherence spectroscopy (HSQC) spectra were recorded using a phase-sensitive, sensitivity-enhanced pulse sequence using echo / anti-echo detection and shaped adiabatic pulses (hsqcetgpsisp2.2) for ^1^H-^13^C HSQC experiments [56]. The experiments were performed at 293 K with a 16.45 T Bruker Avance Neo spectrometer using an inverse triple resonance cryoprobe with an acquisition time of 2 s and a recycle delay of 6 s for the ^1^H experiment, and an acquisition time of 213 ms and recycle delay of 1.75 s for the HSQC experiments.

### Mass spectrometry analysis

Ion chromatograph-mass spectrometry (IC-MS) samples were prepared by dissolving the lyophilized powder of the polar fractions in 18 MΩ water. The metabolites were separated with a Dionex ICS6000 system using modifications to the method described by Sun et al. [57].

The separated metabolites were analyzed using an Orbitrap Fusion Lumos mass spectrometer (Thermo Fisher Scientific). The MS settings were as follows: scan range(m/z) 70-800, resolution 500,000 at m/z 200, RF lens (%) 30, sheath gas flow rate 50 arbitrary units (Arb), auxiliary gas flow at 10 Arb, sweep gas flow at 1 Arb, ion transfer tube temperature 325 °C, vaporizer temperature 350 °C, and negative ion voltage 2 500 V. An in-house metabolite database was incorporated into TraceFinder software (Thermo Fisher Scientific) for peak assignments and peak area measurements. Metabolite standards were prepared in-house and run at different concentrations to establish calibration curves for each metabolite. Following correction for ^13^C natural abundance (PREMISE software kindly provided by Dr. Richard Higashi, University of Kentucky) and the split ratio of the aliquot used for analysis, the calculated amount of each metabolite was further normalized to the protein quantity.

### Metabolomics data analysis

To identify differentially expressed metabolites, a heatmap and volcano plot were created using the Qlucore Omics Explorer (ver. 3.8; Lund, Sweden).

### Survival analysis using The Cancer Genome Atlas (TCGA) database

Genomic and clinical data were obtained from TCGA portal. The results shown here were based on data generated by TCGA Research Network (https://www.cancer.gov/tcga). Patients in high-quartile and low-quartile gene expression groups were identified for NAPRT, QPRT, and NADSYN1 (lower percentile = 25% [*n* = 73], and upper percentile = 25% [*n* = 73]).

### Correlation analysis using DepMap portal data

Correlation analysis was performed using the integrated DepMap Data Explorer tool (https://depmap.org/portal/). Gene expression levels (Expression Public 23Q2 release) and metabolomic abundance data were extracted on September 10, 2023.

### RNA interference

For the short interfering RNA (siRNA) experiments, cells were transfected with a final concentration of 30 nM of ON-TARGETplus Human non-targeting siRNA (#D-001810-01-20, Dharmacon), ON-TARGETplus SMARTpool Human NAMPT (#L-004581-00-0005, Dharmacon), NAPRT1 (#L-016912-01-0005), QPRT (#L-003989-01-0005), and NADSYN1 (#L-007723-01-0005) targeting siRNAs using Lipofectamine RNAiMAX Reagent (#13778-150, Thermo Fisher Scientific) and Opti-MEM I Reduced Serum Medium (#11058-021, Thermo Fisher Scientific) following the manufacturer’s protocol. The following day, the cells were trypsinized and seeded onto 10 cm dishes. Cells were harvested 72 h post-transfection.

### Seahorse bioenergetic assays

Cells were cultured in 96-well ultra-low attachment (ULA) plates (#7007, Corning, NY, USA) with stem cell culture Media containing 0.2 mM Matrigel (#354230, Corning). Cells were treated with reagents, including GNE-617 and disulfiram, for 48 h. Spheroids were transferred to XFe96 Spheroid Microplates (#102978-100, Agilent, CA, USA) and incubated with 175 μl of serum-free unbuffered Seahorse XF RPMI Medium (pH 7.4) with 1 mM HEPES (#103576-100, Agilent) pre-warmed at 37 °C and supplemented with 10 mM glucose, 2 mM glutamine, and 1 mM pyruvate (for analysis of mitochondrial oxidative metabolism). Cartridges equipped with oxygen- and pH-sensitive probes were pre-incubated with a calibration solution (#100840-000, Agilent) overnight at 37 °C in a CO_2_-free incubator. The OCR and ECAR were assessed over time, before and after injecting compounds from the Seahorse XF Cell Mito Stress Test Kit: Oligomycin (1 μM), FCCP (2 μM), and Rotenone + Antimycin (0.5 μM each). The OCR and ECAR values were adjusted based on spheroid area.

### Co-treatment and interaction

Treated cells were incubated for 72 h before cell viability was measured using the CellTiter-Glo Luminescent Cell Viability Assay (#G7571, Promega). Synergistic interactions were quantified using the Combenefit program and the HSA model (https://www.cruk.cam.ac.uk/research-groups/jodrell-group/combenefit).

### ALDH activity measurements

ALDEFLUOR Kit (#01700, STEMCELL Technologies, Vancouver, Canada) were used according to the manufacturer’s protocol. Cells were seeded on to regular 6 well plates (2D) or ULA 6 well plates (3D) with respective media. After 96 h incubation, 2D cells were collected by trypsinization, and 3D-spheroids were collected with the media. Single cell suspension was prepared from 3D-spheroids with TryPLE express enzyme (#12604013, Thermo Fisher Scientific) for 10–20 min incubation at 37 °C. Cell numbers were analyzed by AOPI assay. For flow cytometry experiments, cell suspensions were prepared for each condition, and once fluorescence reagents (ALDEFLUOR reagent), LIVE/DEAD Aqua (#L34957, Thermo Fisher Scientific) were added, the cells were incubated for 45 min at 37 °C. The cells were then washed twice, resuspended with 0.5 ml the assay buffer, and filtered using Cell Strainer (#352235, BD Falcon) spun down at 300 g for 3 min, prior to analysis with a BD FACS Verse Flow Cytometer. LIVE/DEAD Aqua was used to exclude dead cells from analysis. Data were analyzed with FlowJo software (ver. 10.7.2, Becton, Dickinson and Company, NJ, USA).

### GAPDH-mediated response quantification

GAPDH-mediated responses were determined using a glyceraldehyde 3-phosphate Dehydrogenase Activity Assay Kit (#ab204732, Abcam, Cambridge, UK), according to the manufacturer’s instructions. The reaction mixture was added to the extracted samples and incubated at 37 °C for 30 min. NADH levels reflecting GAPDH-mediated responses were quantified. The results were normalized to the protein concentration and measured using a SpectraMax i3.

### Aconitase activity quantification

Aconitase activity was determined using the Aconitase Activity Assay Kit (#ab109712, Abcam) following the manufacturer’s instructions. The results were normalized to the protein concentration and measured using a SpectraMax i3.

### MitoSOX Red intensity measurements

The MitoSOX Mitochondrial Superoxide Indicators (#M36008, Thermo Fisher Scientific) intensity (excitation/emission, 510/580 nm) was measured according to the manufacturer’s instructions. The cells were added to the staining solution at a final concentration of 1 µM and incubated for 30 min at 37 °C. After washing with PBS, fluorescence was measured using a SpectraMax i3. The values were normalized to the total number of viable cells using the CellTiter-Glo Luminescent Cell Viability Assay (#G7570, Promega, WI, USA) in the same wells following the protocol provided with the assay kit.

### Cell cycle analysis

Cells were grown in culture dishes, collected and resuspended in cold PBS at a concentration of 1–2 × 10^6^ cells/ml. 1 ml of cell suspension was pipetted into a Falcon 12 × 75 mm polystyrene tube with snap cap and placed in an ice bath for 15 min. 9 ml ice-cold 70% ethanol was added gradually (total 10 mL per sample), and samples were stored at 4 °C overnight for fixation. On the day of assay, cells fixed in ethanol were spun down by centrifugation (300 g, 5 min), and the ethanol was removed, and resuspended in PBS containing 0.1% Triton-X, 20 µg/ml RNase, 20 µg/mL propidium iodide and incubated at 37 °C for 20 min. Cell cycle analysis was performed with BD FACS Verse Flow Cytometer. Total of approximately 1 × 10^4^ cells were sorted per each condition. Data were analyzed with FlowJo software.

### Animal experiments

Female athymic Nu/Nu mice (5-6 weeks of age) were purchased from Envigo (Madison, WI, USA) and approved for use by the NCI Animal Care and Use Committee (IACUC number MOB-025). Two types of experiments were conducted: one in which tumor cells were injected subcutaneously, and the other in which tumor cells were injected intraperitoneally. Mice injected with cells were randomized and allocated to experimental groups. In both experiments, the maximum observation period was set at 80 days. Mice with growing tumors or signs of imminent death were sacrificed before reaching 80 days, while surviving mice were sacrificed on day 80, at the conclusion of the experiment. The mice were randomized into groups. Xenografts were established using human cell lines through subcutaneous or intraperitoneal inoculation of nude mice with cells resuspended in 100 μl PBS (subcutaneous injection: 1 x 10^6^ cells; intraperitoneal injection: 5 x 10^6^ cells). Tumor length and width were measured using calipers, and tumor volume was calculated using the standard formula: (lengthvhhvj bn × width^2^)/2. GNE-617 was dissolved in a solution of PEG400, H_2_O, and EtOH (6:3:1) via sonication, and a dose of 15 mg/kg was administered at 100 μl/mouse by gavage twice a day at 7 am and 3 pm for five consecutive days. Disulfiram was dissolved in 12.5% Captisol (#RC-0C7-020, Ligand Pharmaceuticals, CA, USA) through sonication and administered intraperitoneally at 200 μl once a day at 7 am for five consecutive days. The mice were monitored daily for health, and survival was recorded on days when the mice met the NIH Animal Care and Use Committee-approved humane criteria for euthanasia.

### Statistical analyses

The number of biological replicates was determined based on alpha error, beta error, and effect size according to standard practices used in this domain of application. Data presented as ± SD. Statistical analyses were performed using the GraphPad Prism 8 software (Boston, MA, USA). For small sample sizes, we initially performed multiple tests to assess normality, including the QQ test, Anderson-Darling test, D’Agostino-Pearson omnibus test, Shapiro-Wilk test, and Kolmogorov-Smirnov test. Experiments that demonstrated high normality were then selected for statistical analysis using Student’s *t*-test and One-way ANOVA for Tukey’s multiple comparisons test, log-rank (Mantel-Cox) test, or Pearson correlation: *p < 0.05, **p < 0.01, ***p < 0.001, ****p < 0.0001.

## Supplementary information

Supplementary items

Supplementary Figure 1

Supplementary Figure 2

Supplementary Figure 3

Supplementary Figure 4

Supplementary Figure 5

Supplementary Figure 6

Supplementary Figure 7

Supplementary Figure 8

Raw western

qPCR primer

## Data Availability

The data generated and/or analyzed during this study are available from the corresponding author upon reasonable request.
