# Supplementary material for: Targeting metabolic vulnerability by combining NAMPT inhibitors and disulfiram for treatment of recurrent ovarian cancer
Source: Cell Death Dis. 2025 Apr 25;16(1):342. doi: 10.1038/s41419-025-07672-3 (PMC12032209; doi:10.1038/s41419-025-07672-3)
Supplement: Supplementary file 1 — Supplementary items [file 41419_2025_7672_MOESM1_ESM.docx]

**Supplemental items**

**Supplementary Figure 1**

NAMPT is a prognostic factor and a potential therapeutic target against 3D-spheroids with cancer stemness features, as related to Figure 1.

**Supplementary Figure 2**

NAMPT inhibition is a potential strategy against NAMPT-dependent OV, as related to Figure 1.

**Supplementary Figure 3**

3D-spheroids oxidize glucose via the TCA cycle for elevated ATP production, related to Figures 3.

**Supplementary Figure 4**

3D-spheroids enhance the activity of the PC-mediated anaplerotic TCA cycle and *de novo* pyrimidine synthesis, as related to Figure 3 and 4.

**Supplementary Figure 5**

The combination of NAMPT inhibitors and disulfiram inhibits cell viability, as related to Figure 5.

**Supplementary Figure 6**

Disulfiram and combinatorial treatment suppress glucose oxidation and the anaplerotic TCA cycle, as related to Figure 6.

**Supplementary Figure 7**

Combinatorial treatment induces a synergistic anti-tumor effect by suppressing mitochondrial function and impairs cell viability, as related to Figure 7.

**Supplementary Figure 8**

Combinatorial treatment significantly inhibits tumor growth and extends the survival *in vivo*, as related to Figure 8.

**Supplementary Figure legends**

**Supplementary Figure 1. NAMPT is a prognostic factor and a potential therapeutic target against 3D-spheroids with cancer stemness features**

**(A)** Schematic illustrating the process of obtaining 2D- and 3D-cultured cells.

**(B)** qPCR detection of representative stem cell marker genes comparing 2D- and 3D-cultured cells in A2780 and IGROV1 cells (n = 4 - 8 independent experiments).

**(C)** Top: Immunoblotting to assess representative stemness marker CD133 in 2D- and 3D-cultured A2780 and IGROV1 cells. GAPDH was used as a control. Bottom: The protein levels were normalized to GAPDH (*n* = 3 independent experiments).

**(D)** Comparison of ALDH activity between 2D- and 3D-cultured A2780 and IGROV1 cells was conducted using ALDEFLUOR fluorescence, measured as geometric MFI (n = 3 independent experiments).

**(E)** Schematic representation of the NAD^+^ production pathway.

**(F)** Kaplan-Meier curves depicting the overall survival of OV patients with either low (blue) or high (red) levels of NAPRT, QPRT, and NADSYN1 proteins, based on TCGA data.

**(G)** Correlation analysis between the gene expressions of NAPRT, QPRT, and NADSYN1 (Public 23Q2) and levels of total NAD in OV or fallopian cancer cell lines, based on data from the DepMap portal.

**(H)** Immunoblotting to evaluate the silencing of enzymes involved in NAD^+^ production in 2D-cultured A2780 and IGROV1 cells. HSP90 was used as a control.

**(I)** Comparison of total NAD levels normalized to protein concentration in the same cells as in (H). (*n* = 3 independent experiments)

**(J)** Basal respiration, proton leak, and Spare respiratory capacity in OCR (*n* = 5 independent experiments).

**(K)** Left: Representative OCR pattern over time (min) in the same cells as in (H), normalized to the cell count. Oligomycin (Oligo), carbonyl cyanide-4-(trifluoromethoxy)phenylhydrazone (FCCP), rotenone, and antimycin A (R/A) were added to measure Basal OCR, ATP content, maximal OCR, and Non-mitochondrial OCR. (*n* = 5 technical replicates) Middle: Maximal respiratory capacity in OCR (*n* = 5 technical replicates) Right: Maximal glycolytic capacity in ECAR (*n* = 5 technical replicates)

Graph data were presented as mean ± SD of multiple experiments.

**Supplementary Figure 2. NAMPT inhibition is a potential strategy against NAMPT-dependent OV**

**(A)** Schematic diagram depicts the addition of NMN or NA to stem cell culture media to assess the effect of NMN or NA rescue.

**(B)** Schematic diagram illustrates that treatment with NAMPT inhibitor induces cell death in NAD^+^-dependent cell lines, even when NA is present in the media. The same treatment for cell lines that also synthesize NAD^+^ from NA results in cell growth (NA rescue).

**Supplementary Figure 3. 3D-spheroids oxidize glucose via the TCA cycle for elevated ATP production**

**(A)** Comparison of cell growth between 2D- and 3D-cultured A2780 and IGROV1 cells with AOPI assay (n = 4 - 6 independent experiments). These cells were cultured in either 2D- or 3D-medium.

**(B)** Comparison of cell viability between 2D- and 3D-cultured A2780 and IGROV1 cells with MTS cell viability assay (n = 4 independent experiments). These cells were cultured in the same 3D-medium.

**(C)** Kinetic graphs showing cell proliferation in 2D-cultured IGROV1 and A2780 cells, with the medium of the indicated composition over time (hours) (*n* = 4 independent experiments)

**(D)** Schematic of combined approaches using nuclear magnetic resonance (NMR) and ion chromatography-mass spectrometry (IC-MS) to assess the role of ^13^C_6_-glucose in central carbon metabolism, comparing 2D- and 3D-cultured cells.

**(E)** 1D ^1^H-^13^C spectra obtained from 2D-cultured (red) and 3D-cultured (blue) IGROV1 cells grown in the presence of ^13^C_6_-glucose for 24 h, as compared to 2D-cultured cells grown in the presence of non-^13^C-labeled natural abundance glucose (gray). Chemical shift assignments for the 1D ^1^H-^13^C HSQC spectra are based on the combined information acquired from ^1^H-^1^H TOCSY, 2D HSQC-TOCSY experiments, as well as IC-MS analyses, all performed on the same samples. These combined analyses revealed that the majority of the UDP-glucose resonances observed in the ^1^H-^13^C HSQC spectrum of 3D-cultured cells were in the form of UDP-N-acetylglucosamine (UDP-GlcNac).

**(F)** Comparison of the glucose consumption in 2D- and 3D-cultured cells after 24 h incubation. (*n* = 3 independent experiments)

**(G)** Schematic illustrating showing HK-mediated conversion of glucose to G6P.

**(H)** Left: Immunoblotting conducted to assess the levels of HK2 in both 2D- and 3D-cultured IGROV1 cell lines, with HSP90 serving as a control. Right: Comparison of HK2 levels, normalized to HSP90 levels (*n* = 3 independent experiments).

AXP: AMP+ADP+ATP, UXP: UMP+UDP+UTP, G6P: glucose-6-phosphate, UDP: uridine diphosphate, HK: hexokinase

Graph data were presented as mean ± SD of multiple experiments.

**Supplementary Figure 4. 3D-spheroids enhance the activity of the PC-mediated anaplerotic TCA cycle and *de novo* pyrimidine synthesis**

**(A)** Schematic of cataplerotic input of ^13^C_6_-glucose. Labeled ^13^C carbons are shown as colored circles, and unlabeled ^12^C carbons as white circles. Labeling in the first turn of the TCA cycle is by black circles, and in the second turn by gray circles.

**(B)** Schematic of anaplerotic input from ^13^C_6_-glucose. Labeled ^13^C carbons are shown as colored circles, and unlabeled ^12^C carbons as white circles. Labeling in the first turn of the TCA cycle is represented by black circles, and in the second turn by gray circles.

**(C)** Left: Immunoblotting conducted to assess the levels of PC in both 2D- and 3D-cultured A2780 and IGROV1 cell lines, with HSP90 serving as a control. Right: Comparison of PC levels, normalized to HSP90 levels (*n* = 3 independent experiments).

**(D)** Comparison of key metabolite levels in *de novo* pyrimidine synthesis in 2D- and 3D-cultured IGROV1 cells. The carbon distribution of the metabolites is also illustrated (n = 3 independent experiments).

G6P: glucose-6-phosphate, F6P: fructose-6-phosphate, 3PG_2PG: 3-phosphoglycerate or 2-phosphoglycerate, PEP: phosphoenolpyruvate, α-KG: alpha-ketoglutarate, PC: pyruvate carboxylase, PDH: pyruvate dehydrogenase, PRPP: phosphoribosylpyrophosphate

Graph data were presented as mean ± SD of multiple experiments.

**Supplementary Figure 5. The combination of NAMPT inhibitors and disulfiram inhibits cell viability**

**(A)** HSA values generated by Combenefit showing the analysis of the interaction between disulfiram and GNE-617, FK-866, GNE-618, and KPT-9274 in 3D-cultured A2780 and IGROV1 cells. (*n* = 4 independent experiments)

**(B)** Synergy plots generated by Combenefit illustrating the interaction between disulfiram and FK-866, GNE-618, and KPT-9274 in 3D-cultured A2780 and IGROV1 cells. (*n* = 4 independent experiments)

**(C)** Representative figures depicting changes in ALDH activity (ALDEFLUOR) in 3D-cultured A2780 and IGROV1 cells with or without DEAB. Cells enclosed in the trapezoid represent ALDEFLUOR-positive cells.

Graph data were presented as mean ± SD of multiple experiments.

**Supplementary Figure 6. Disulfiram and combinatorial treatment suppress glucose oxidation and the anaplerotic TCA cycle**

**(A)** Schematic of combined approaches using NMR and IC-MS to evaluate the impact of GNE-617, disulfiram, and their combinatorial treatment on the ^13^C_6_-glucose metabolism in 3D-cultured IGROV1 cells.

**(B)** Principal component analysis (PCA) illustrating robust separation among the groups. Each point in the plot corresponds to an individual sample.

**(C)** Schematic illustrating the change in carbon distribution within the glycolytic and TCA cycle in 3D-cultured IGROV1 cells, as described above. (*n* = 3 independent experiments)

Red circles indicate carbon. "m+0" represents the carbon contribution from sources other than ^13^C_6_-glucose, while "m+1," "m+2," etc. represent the carbon contribution from ^13^C_6_-glucose.

G6P: glucose-6-phosphate, F6P: fructose-6-phosphate, 3PG_2PG: 3-phosphoglycerate or 2-phosphoglycerate, PEP: phosphoenolpyruvate, α-KG: alpha-ketoglutarate, PC: pyruvate carboxylase.

Graph data were presented as mean ± SD of multiple experiments.

**Supplementary Figure 7. Combinatorial treatment induces a synergistic anti-tumor effect by suppressing mitochondrial function and impairs cell viability**

**(A)** Basal respiration, proton leak, and spare respiratory capacity in OCR were measured in 3D-cultured IGROV1 cells treated with control, GNE-617, disulfiram, or a combination of both at the indicated doses for 72 h (n = 8 technical replicates).

**(B)** Top: Immunoblotting to assess the expression of ETC proteins (Complex III and V) in 3D-cultured IGROV1 cell lysates. HSP90 was used as a control. Bottom: Changes in ETC Complex V protein levels, normalized to HSP90, in 3D-cultured IGROV1 cells, as described above (*n* = 3 independent experiments).

**(C)** Left: Representative figures showing changes in ROS production from mitochondria using MitoSOX Red (YFP) in 3D-cultured IGROV1 cells with disulfiram at the indicated dose for 48 h. Cells within the rectangle represent MitoSOX Red-positive cells. Right: Comparison of MitoSOX Red fluorescence using geometric MFI in 3D-cultured IGROV1 cells, as described above. (*n* = 3 independent experiments)

**(D)** Comparison of MitoSOX Red fluorescence using geometric MFI in 3D-cultured A2780 cells with control, GNE-617, disulfiram, and combinatorial treatment at the indicated doses for 48 h. (*n* = 4 independent experiments)

**(E)** Relative cell viability (luminescence reading of RealTime-Glo MT) and cytotoxicity (fluorescence reading of CellTox Green) were measured in 3D-cultured A2780 and IGROV1 cells treated with either DMSO control or the combination therapy (GNE-617 30 nM, disulfiram 600 nM). Z-VAD-FMK (final 20 μM), Nec1 (final 50 μM), or ferrostatin (final 10 μM) was added to the culture 1 hour prior to drug treatment (*n* = 4 or 5 independent experiments).

Graph data were presented as mean ± SD of multiple experiments.

**Figure 8. Combinatorial treatment significantly inhibits tumor growth and extends the survival *in vivo***

**(A)** Comparison of body weight in mice subcutaneously injected with IGROV1 cells before treatment. (*n* = 8 independent experiments)

**(B)** Change in body weight in mice receiving vehicle control, GNE-617, disulfiram, or the combinatorial treatment for 5 consecutive days. (*n* = 8 independent experiments)

**(C)** Comparison of body weight in mice intraperitoneally injected with A2780 cells before treatment. (*n* = 11 independent experiments)

**(D)** Change in body weight in mice receiving vehicle control, GNE-617, disulfiram, or the combinatorial treatment for 5 consecutive days. (*n* = 11 independent experiments)

Graph data were presented as mean ± SD of multiple experiments.
