# Supplementary figures and images for: Targeting metabolic vulnerability by combining NAMPT inhibitors and disulfiram for treatment of recurrent ovarian cancer
Source: Cell Death Dis. 2025 Apr 25;16(1):342. doi: 10.1038/s41419-025-07672-3 (PMC12032209; doi:10.1038/s41419-025-07672-3)

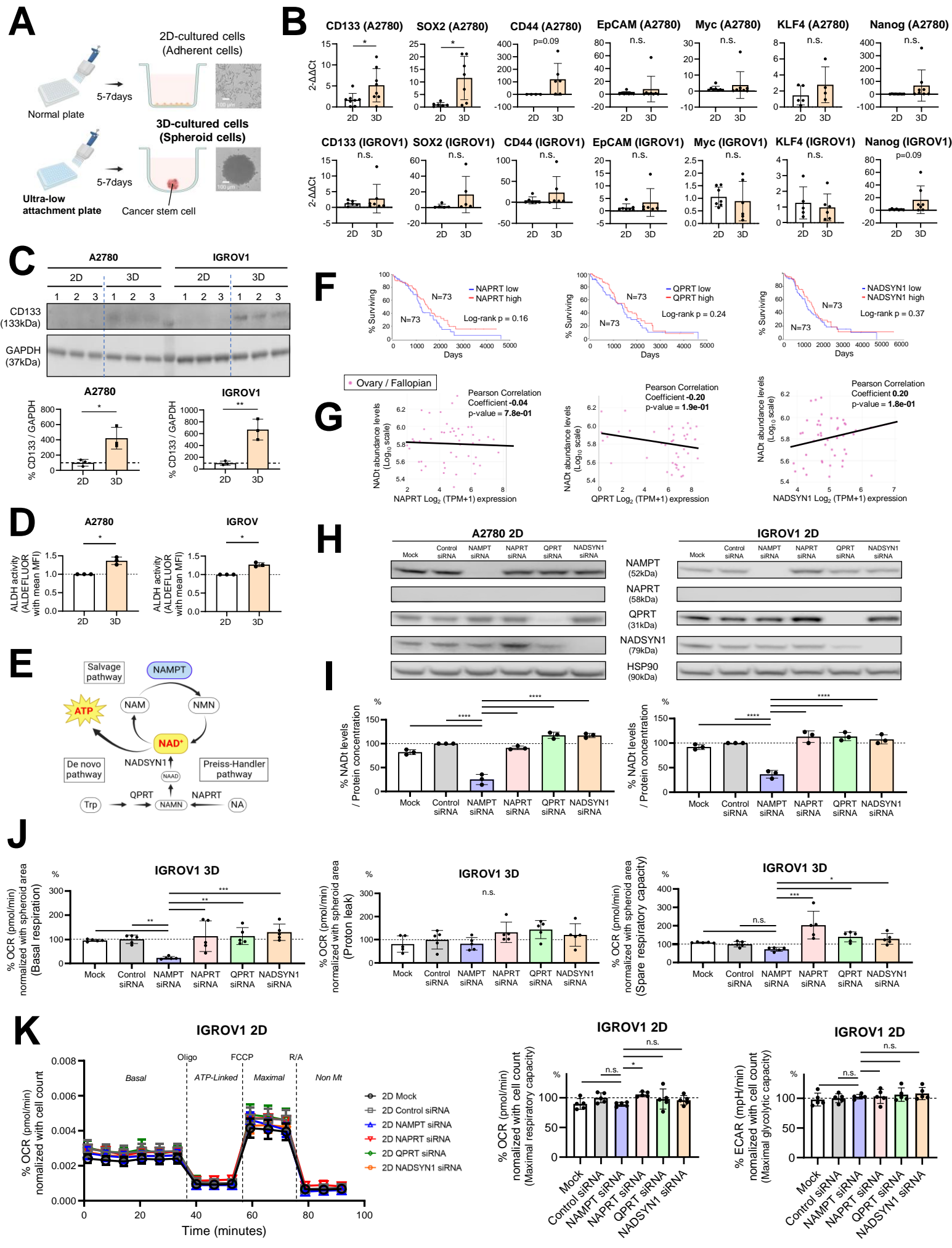

Supplement: Supplementary file 2 — Supplementary Figure 1 [file 41419_2025_7672_MOESM2_ESM.pdf]

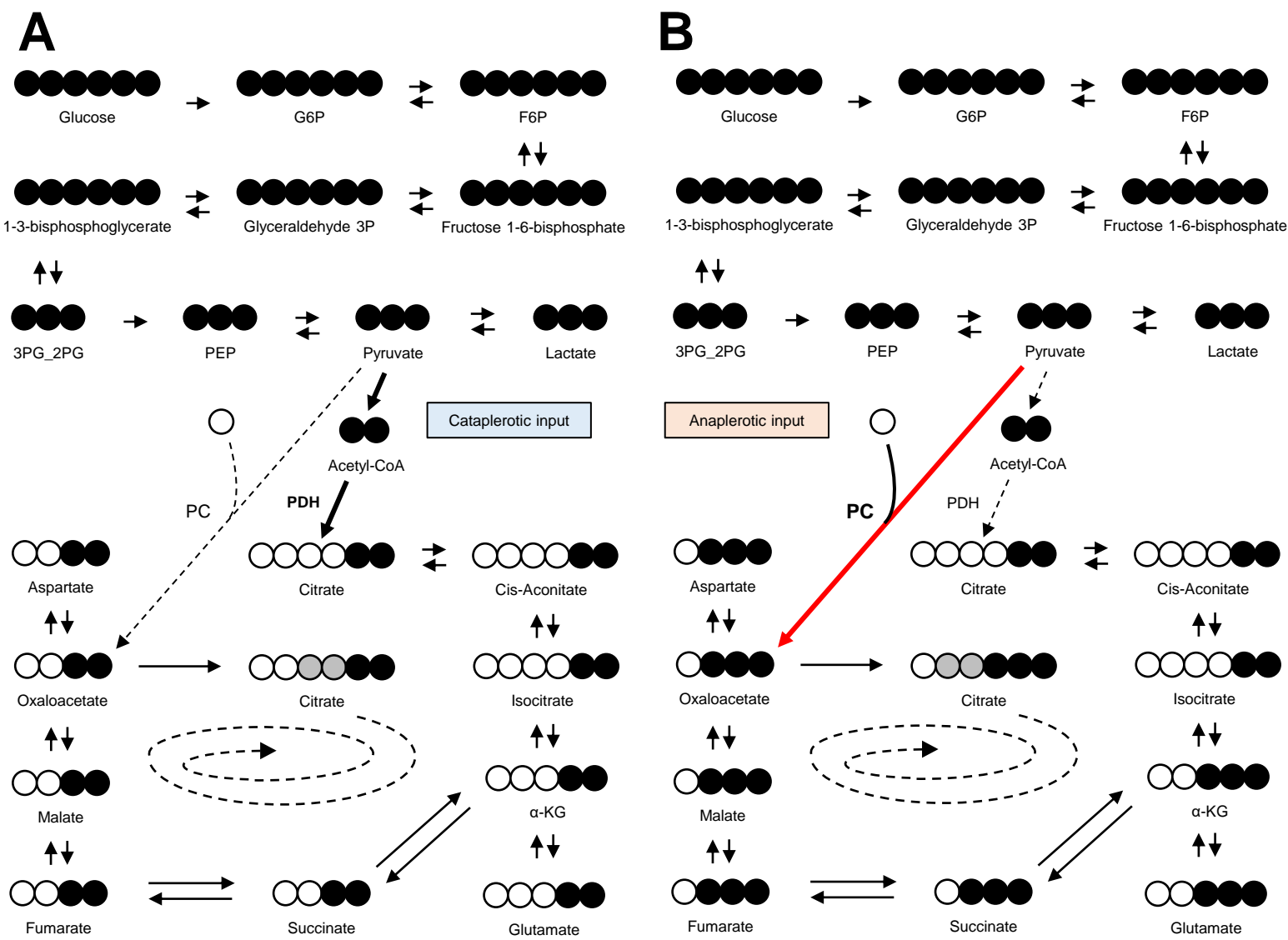

**C**

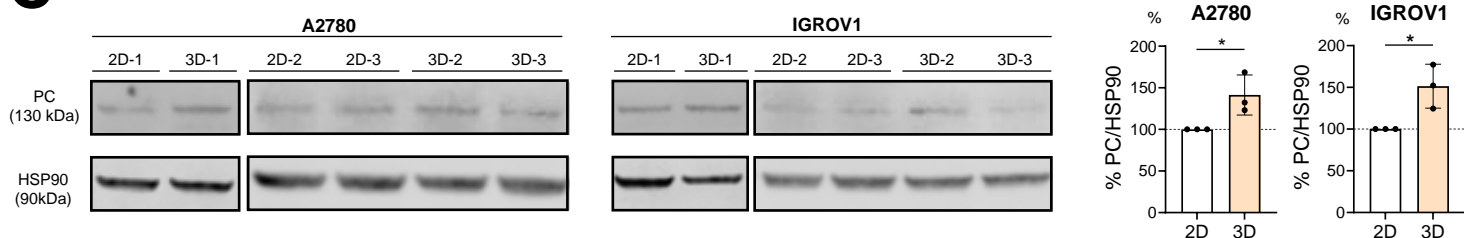

**D**

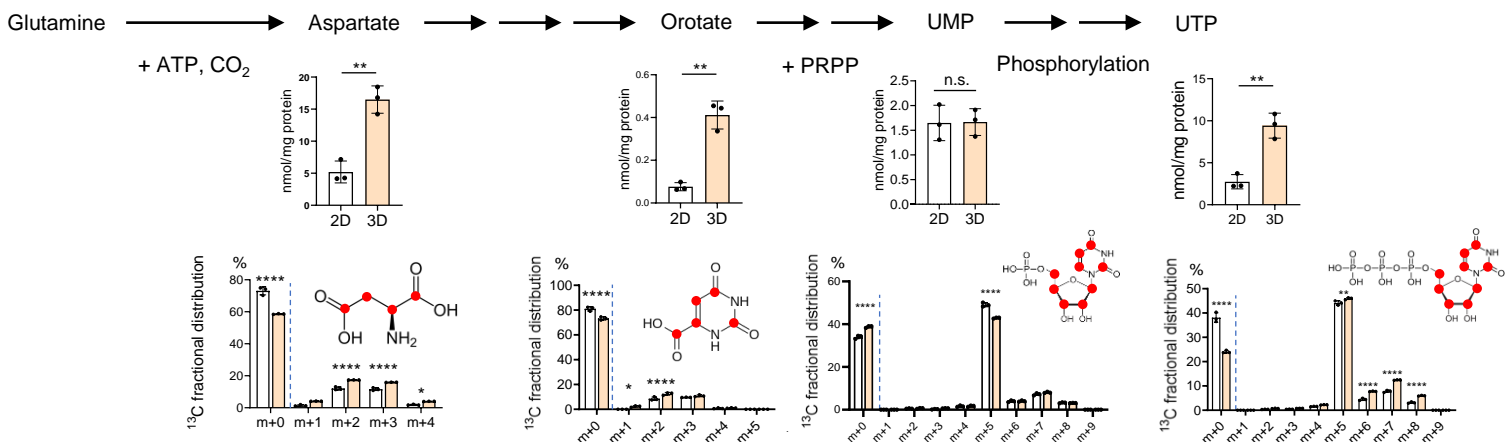

Supplement: Supplementary file 5 — Supplementary Figure 4 [file 41419_2025_7672_MOESM5_ESM.pdf]

# A

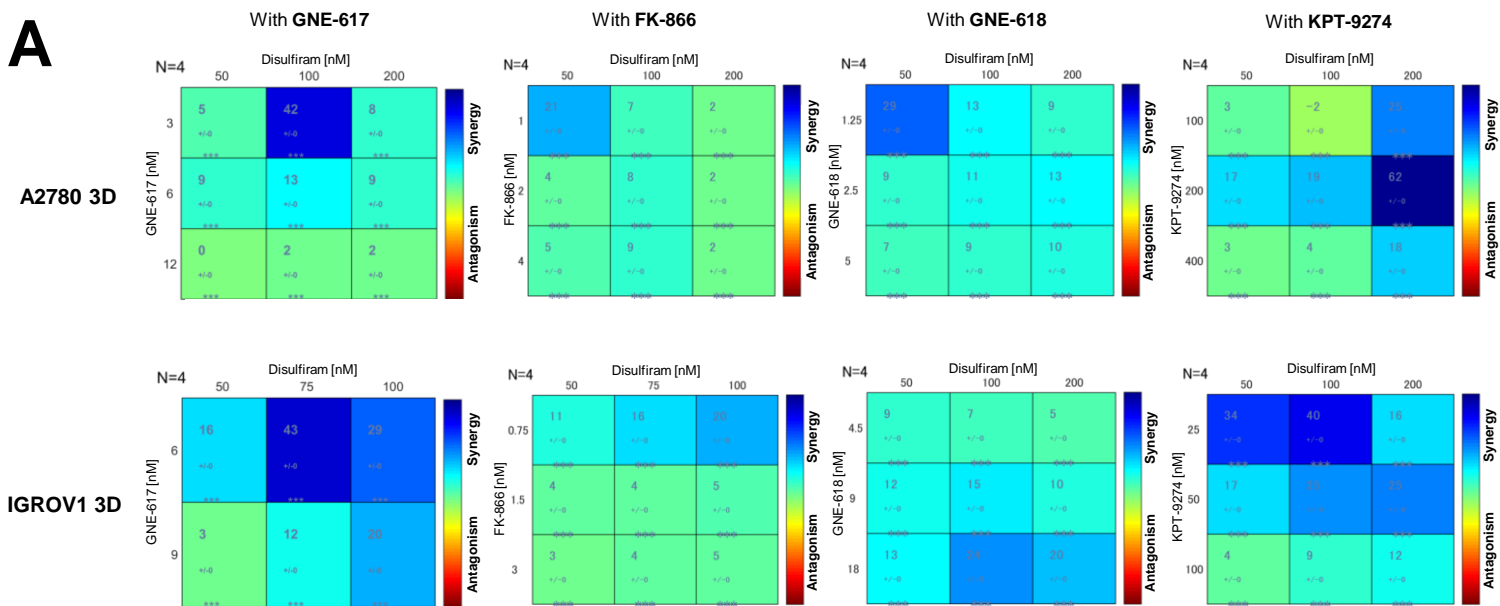

# B

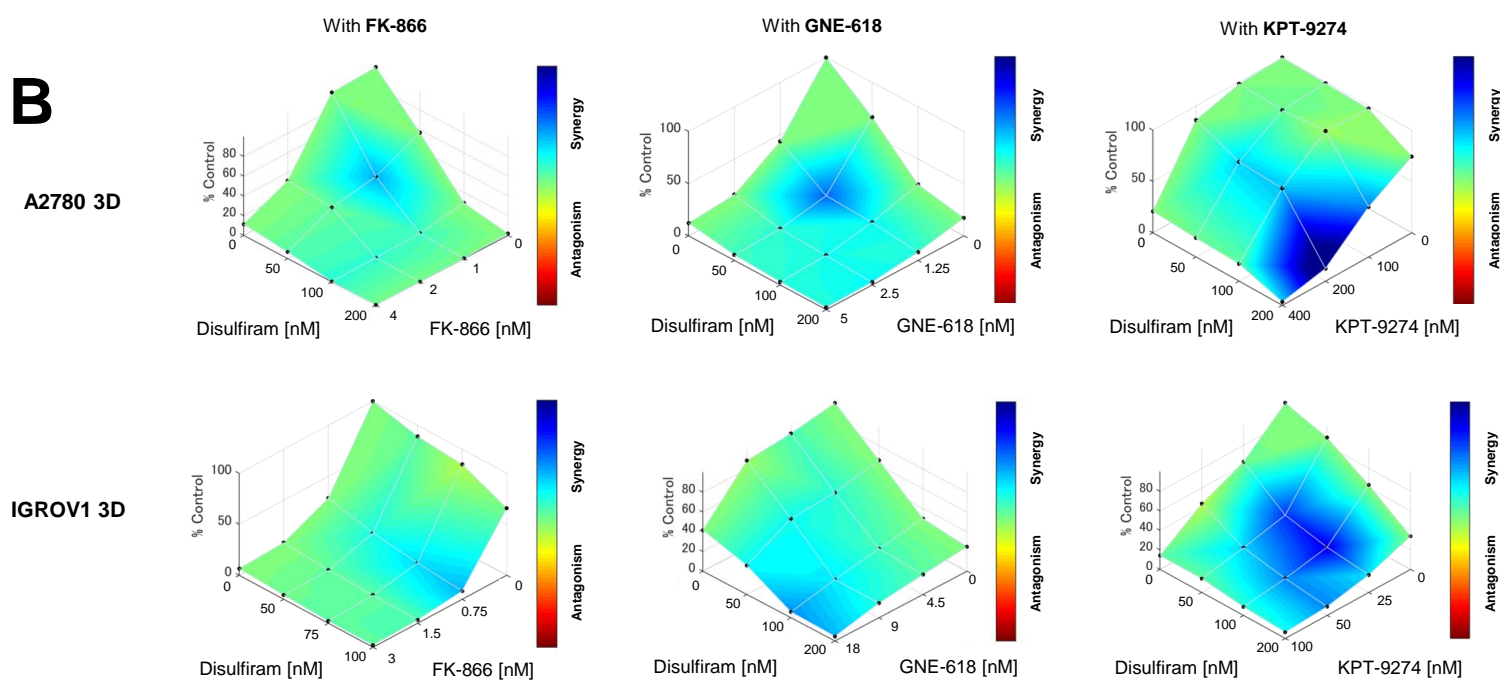

# C

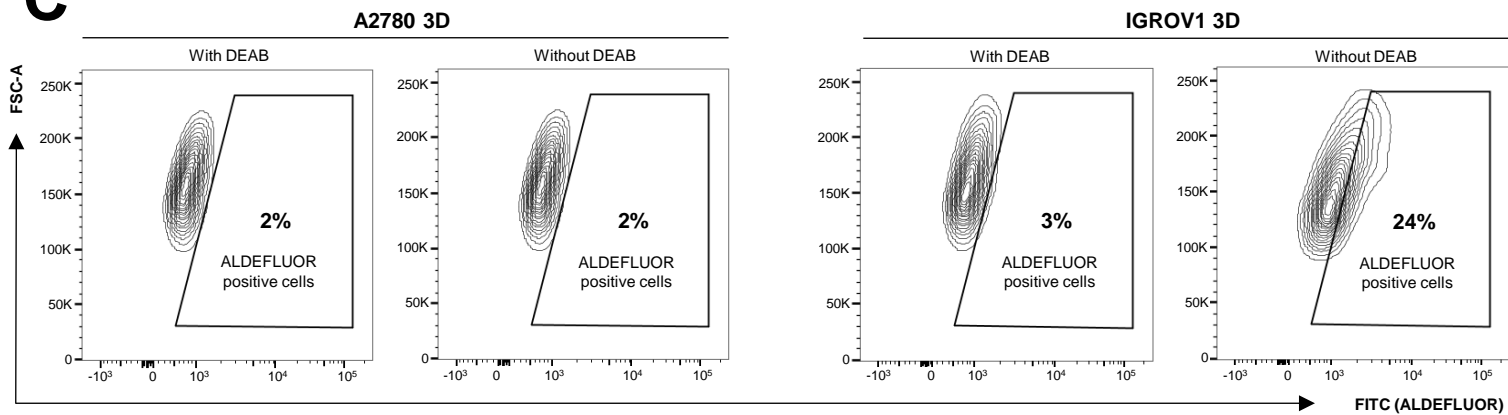

Supplement: Supplementary file 6 — Supplementary Figure 5 [file 41419_2025_7672_MOESM6_ESM.pdf]

**A**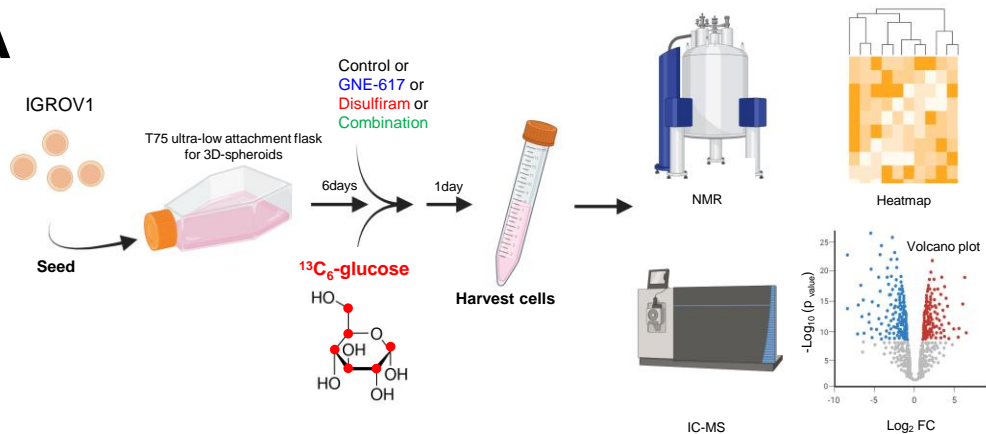**B**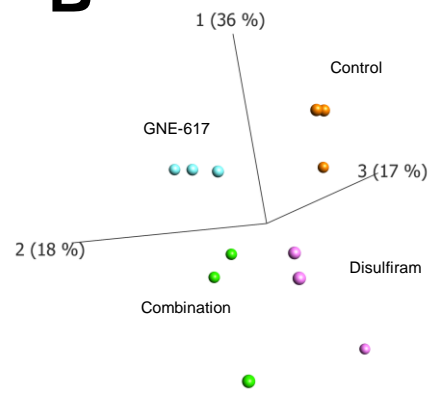**C****Glycolysis**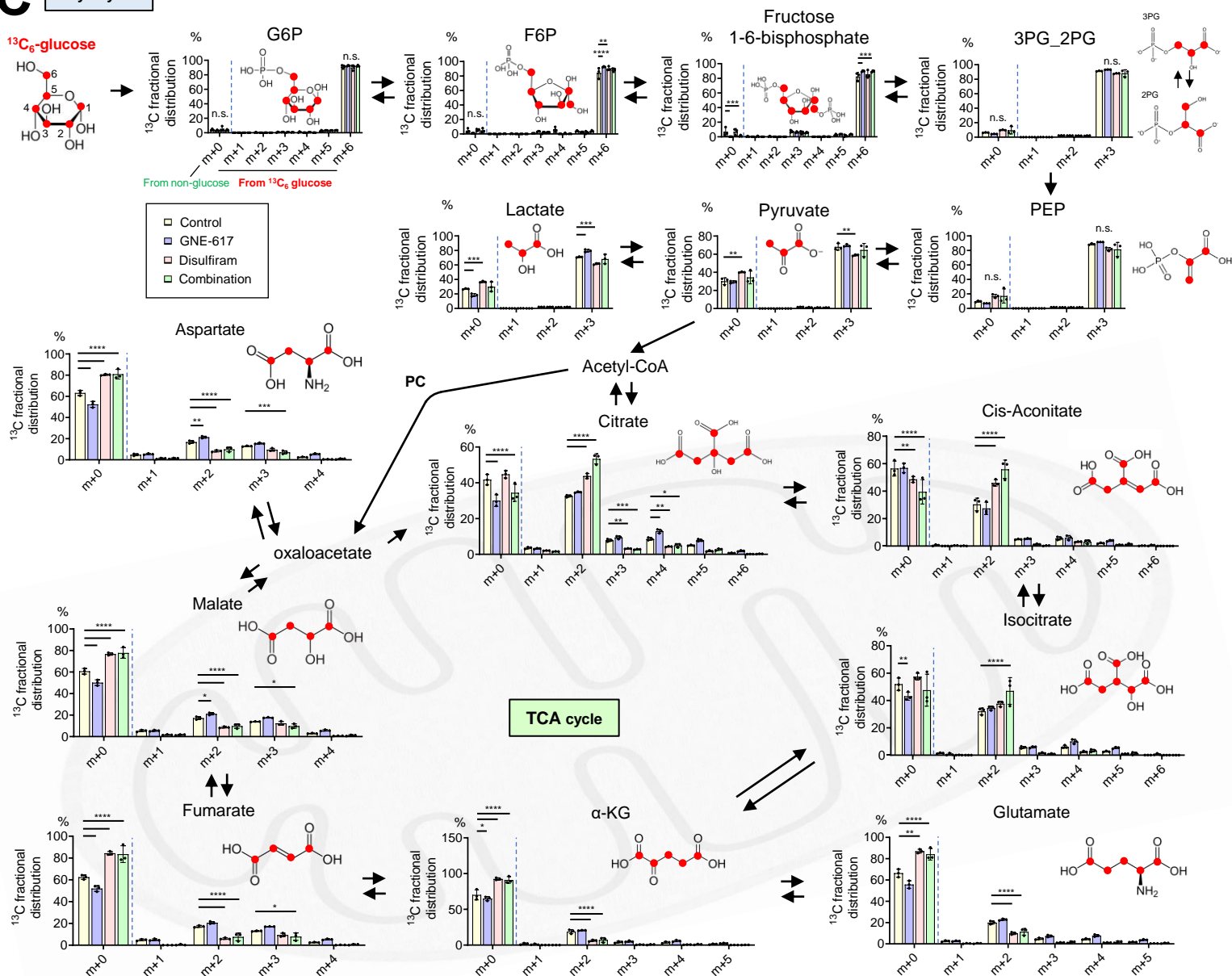

Supplement: Supplementary file 7 — Supplementary Figure 6 [file 41419_2025_7672_MOESM7_ESM.pdf]

**A**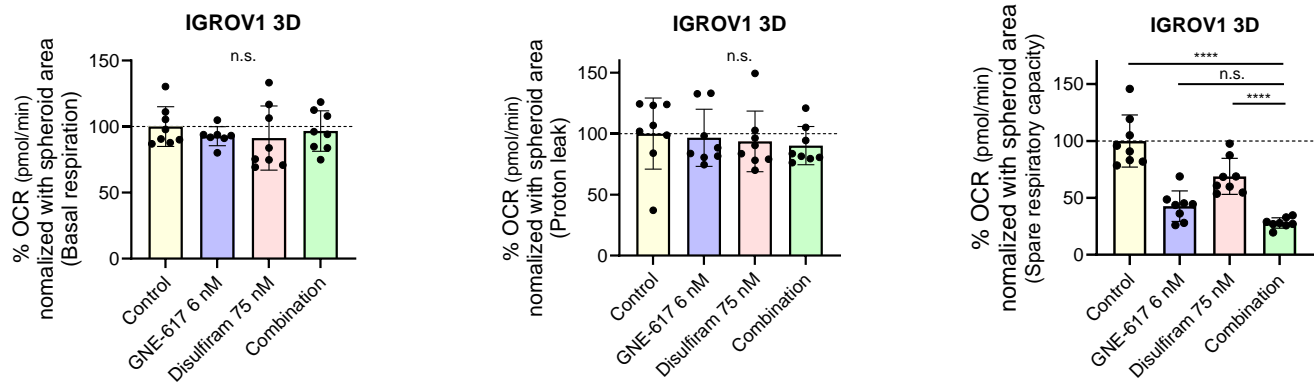**B**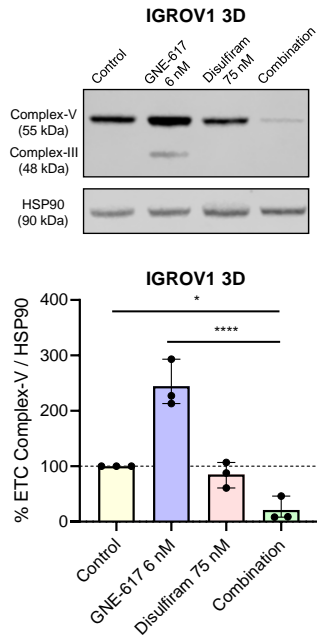**C**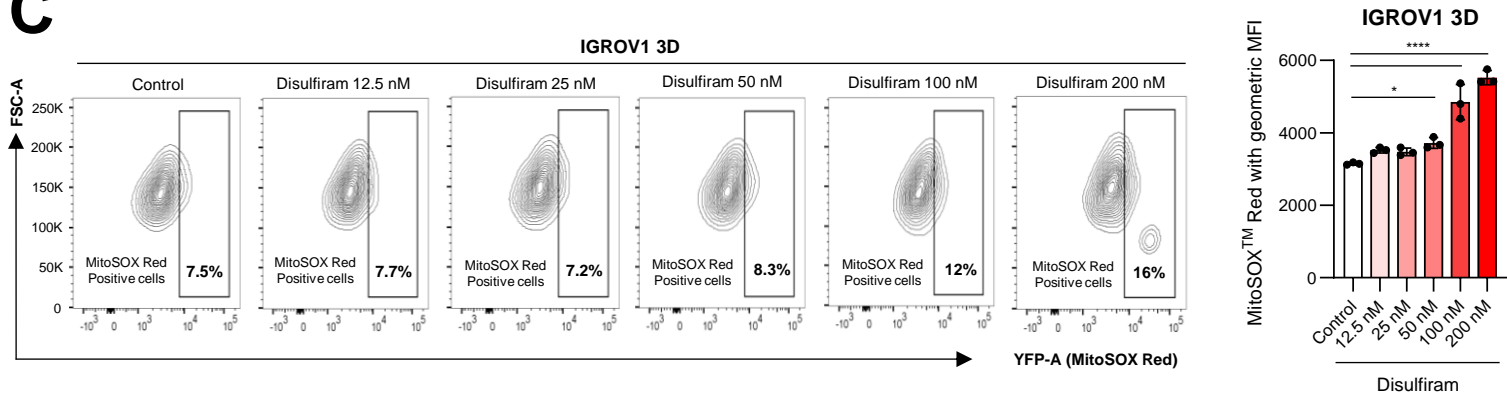**D**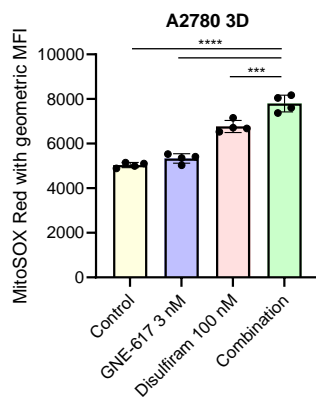**E**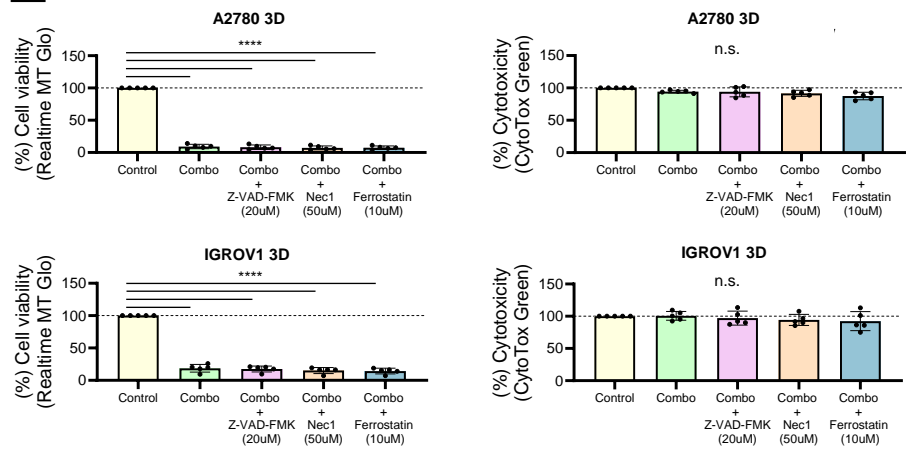

Supplement: Supplementary file 8 — Supplementary Figure 7 [file 41419_2025_7672_MOESM8_ESM.pdf]

# A

## Subcutaneous injection

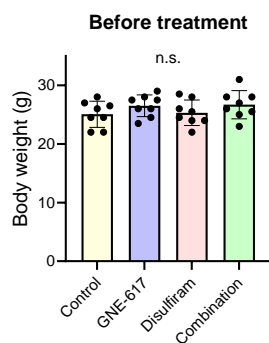

# B

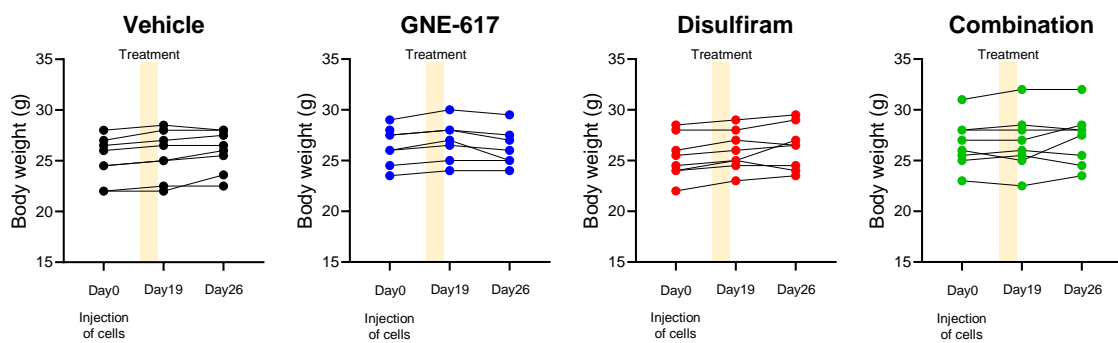

# C

## Intraperitoneal injection

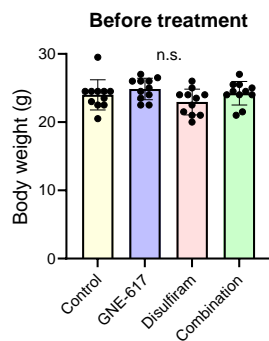

# D

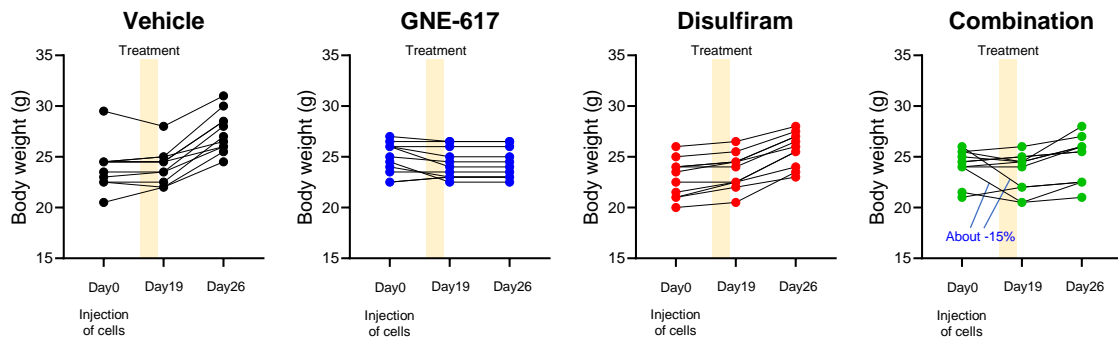

Supplement: Supplementary file 9 — Supplementary Figure 8 [file 41419_2025_7672_MOESM9_ESM.pdf]
