# Supplementary material for: Targeting metabolic vulnerability by combining NAMPT inhibitors and disulfiram for treatment of recurrent ovarian cancer
Source: Cell Death Dis. 2025 Apr 25;16(1):342. doi: 10.1038/s41419-025-07672-3 (PMC12032209; doi:10.1038/s41419-025-07672-3)
Supplement: Supplementary file 3 — Supplementary Figure 2 [file 41419_2025_7672_MOESM3_ESM.pdf]

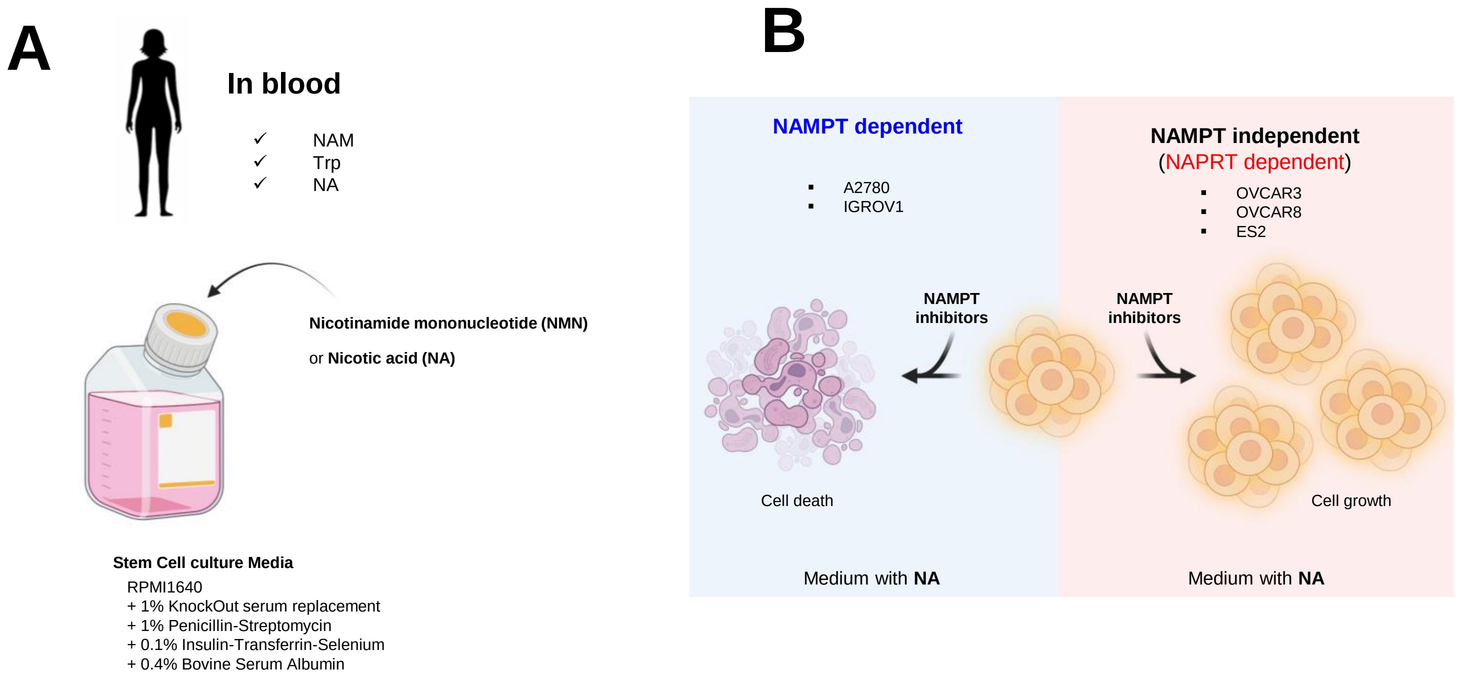
A
In blood
NAM
Trp
NA
Nicotinamide mononucleotide (NMN)
or Nicotic acid (NA)
Stem Cell culture Media
RPMI1640
+ 1% KnockOut serum replacement
+ 1% Penicillin-Streptomycin
+ 0.1% Insulin-Transferrin-Selenium
+ 0.4% Bovine Serum Albumin
B
NAMPT dependent
A2780
IGROV1
NAMPT inhibitors
Cell death
Medium with NA
NAMPT independent
(NAPRT dependent)
OVCAR3
OVCAR8
ES2
NAMPT inhibitors
Cell growth
Medium with NA
