# Supplementary material for: Targeting metabolic vulnerability by combining NAMPT inhibitors and disulfiram for treatment of recurrent ovarian cancer
Source: Cell Death Dis. 2025 Apr 25;16(1):342. doi: 10.1038/s41419-025-07672-3 (PMC12032209; doi:10.1038/s41419-025-07672-3)
Supplement: Supplementary file 4 — Supplementary Figure 3 [file 41419_2025_7672_MOESM4_ESM.pdf]

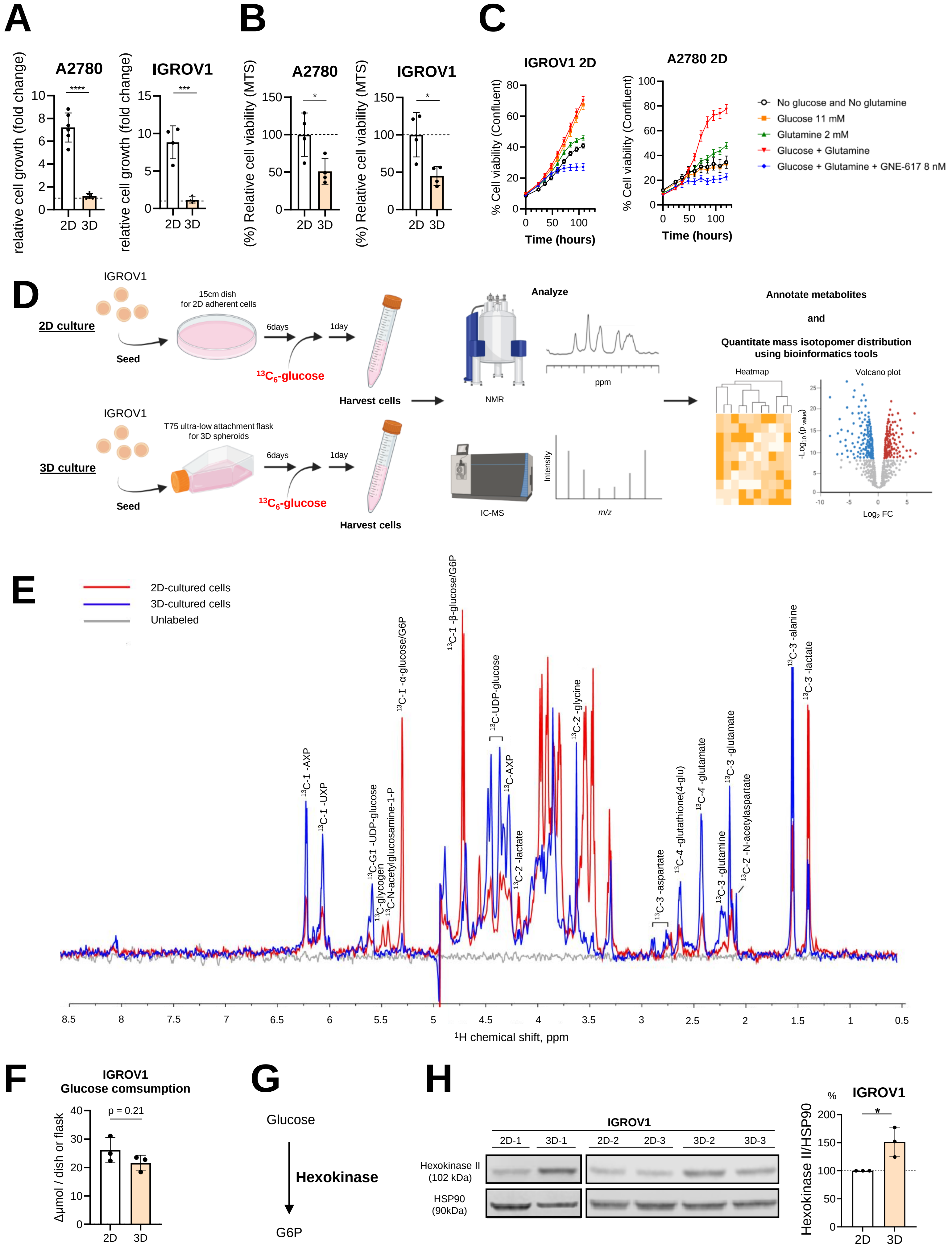

A
A2780
IGROV1
relative cell growth (fold change)
2D 3D
B
(%) Relative cell viability (MTS)
C
IGROV1 2D
A2780 2D
% Cell viability (Confluent)
Time (hours)
No glucose and No glutamine
Glucose 11 mM
Glutamine 2 mM
Glucose + Glutamine
Glucose + Glutamine + GNE-617 8 nM
D
IGROV1
2D culture
15cm dish for 2D adherent cells
Seed
6days
1day
13C6-glucose
Harvest cells
3D culture
T75 ultra-low attachment flask for 3D spheroids
Analyze
NMR
ppm
IC-MS
Intensity
m/z
Annotate metabolites
and
Quantitate mass isotopomer distribution using bioinformatics tools
Heatmap
Volcano plot
-Log10 (p value)
Log2 FC
E
2D-cultured cells
3D-cultured cells
Unlabeled
13C-1 -AXP
13C-1 -UXP
13C-G1 -UDP-glucose
13C-glycogen
13C-N-acetylglucosamine-1-P
13C-1 -α-glucose/G6P
13C-1 -β-glucose/G6P
13C-UDP-glucose
13C-AXP
13C-2 -lactate
13C-2 -glycine
13C-3 -aspartate
13C-4 -glutathione(4-glu)
13C-4 -glutamate
13C-3 -glutamine
13C-3 -glutamate
13C-2 -N-acetylaspartate
13C-3 -alanine
13C-3 -lactate
1H chemical shift, ppm
F
IGROV1
Glucose comsumption
p = 0.21
Δμmol / dish or flask
G
Glucose
Hexokinase
G6P
H
IGROV1
2D-1 3D-1 2D-2 2D-3 3D-2 3D-3
Hexokinase II (102 kDa)
HSP90 (90kDa)
Hexokinase II/HSP90 %
