# Supplementary material for: Targeting metabolic vulnerability by combining NAMPT inhibitors and disulfiram for treatment of recurrent ovarian cancer
Source: Cell Death Dis. 2025 Apr 25;16(1):342. doi: 10.1038/s41419-025-07672-3 (PMC12032209; doi:10.1038/s41419-025-07672-3)

Figure 1

D

IP: NAMPT (52 kDa)

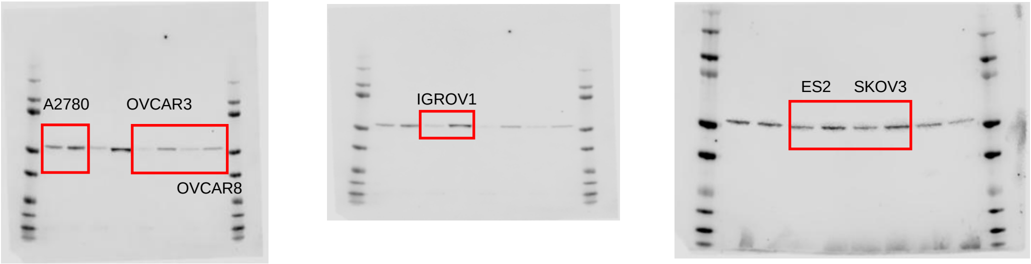

IP: NAPRT (58 kDa)

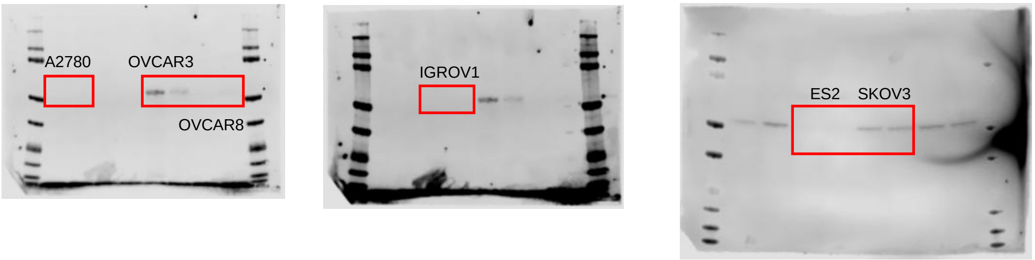

IP: QPRT (31 kDa)

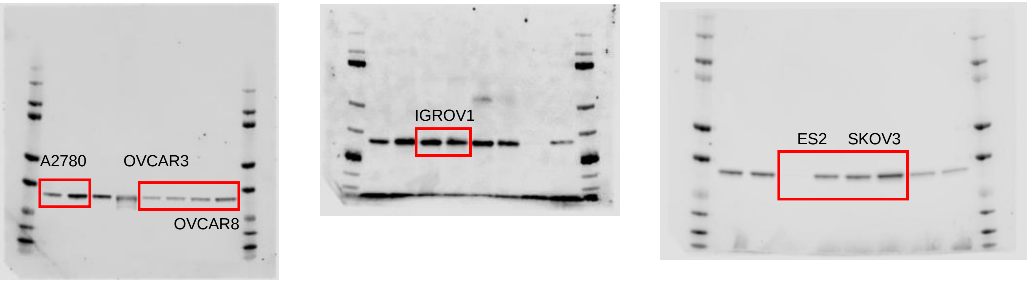

IP: NADSYN1 (79 kDa)

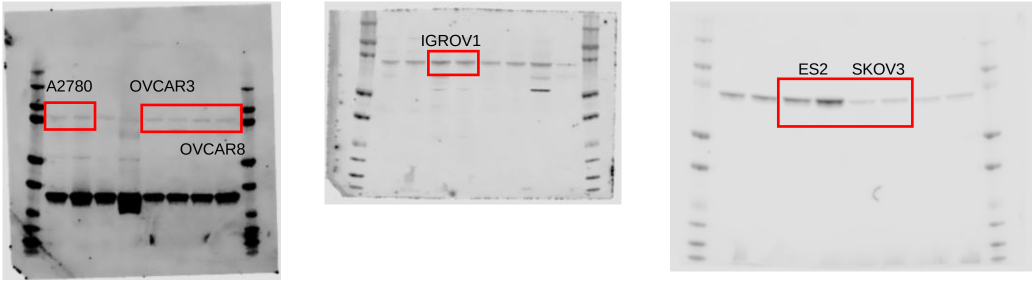

IP: HSP90 (90 kDa)

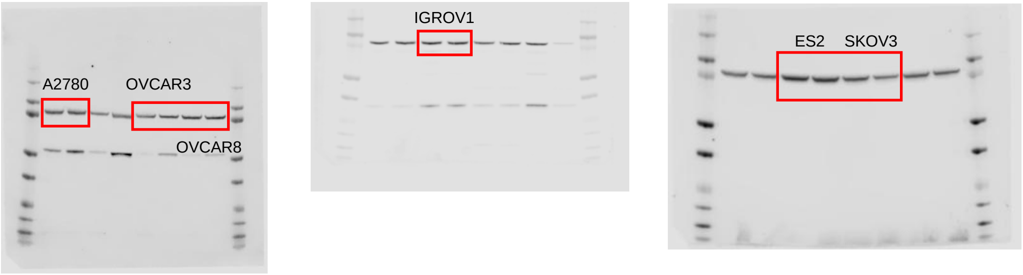

E

IP: NAMPT (52 kDa)

IP: HSP90 (90 kDa)

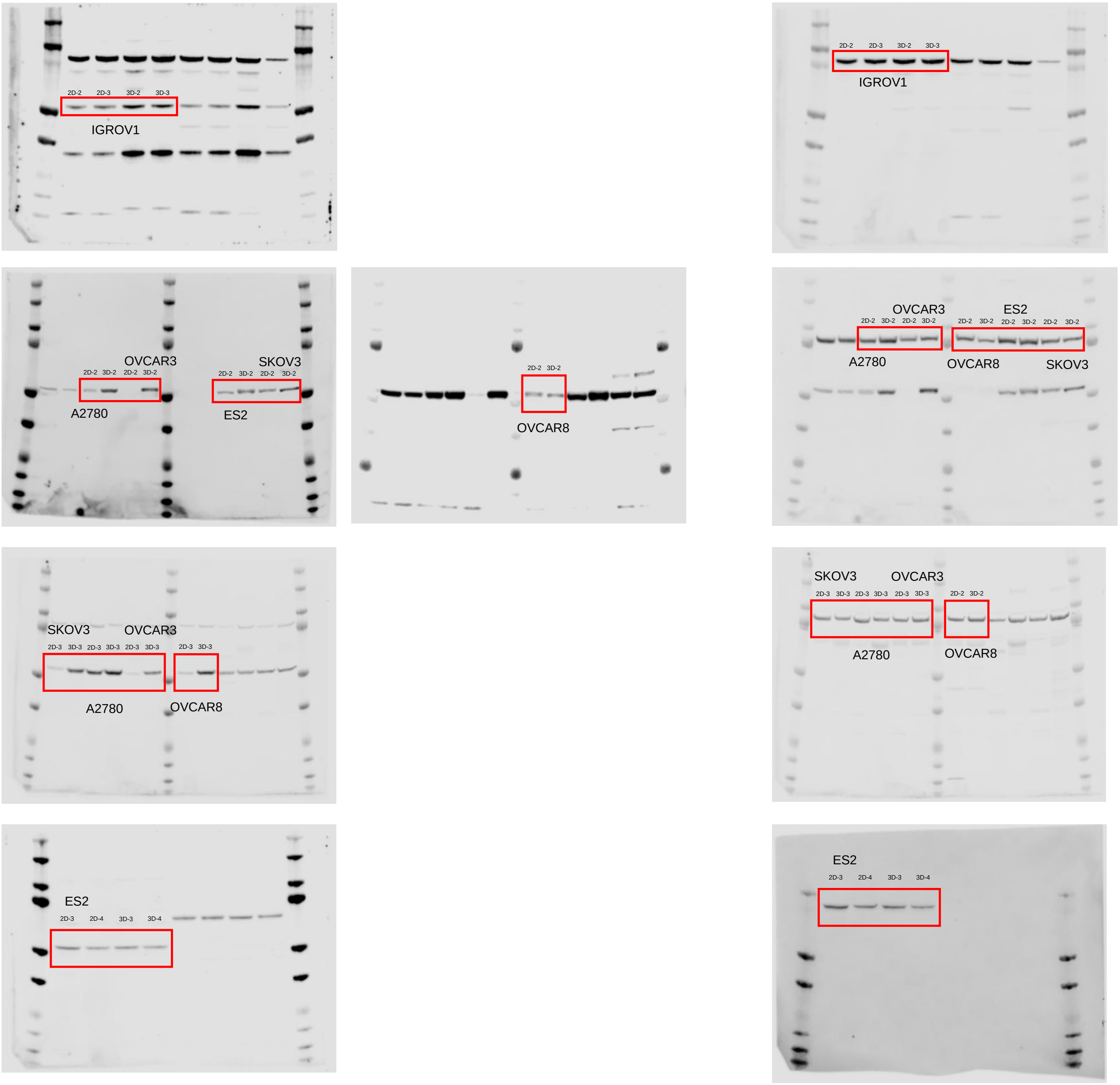

F

IP: NAMPT (52 kDa)

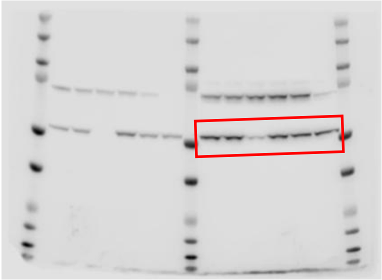

IP: NAPRT (58 kDa)

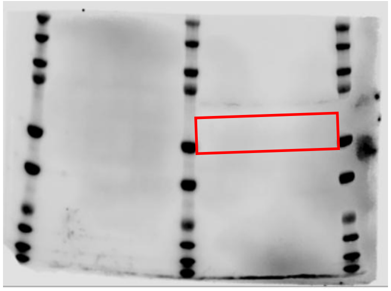

IP: QPRT (31 kDa)

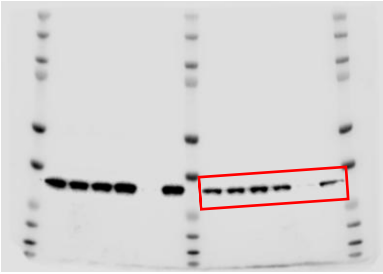

IP: NADSYN1 (79 kDa)

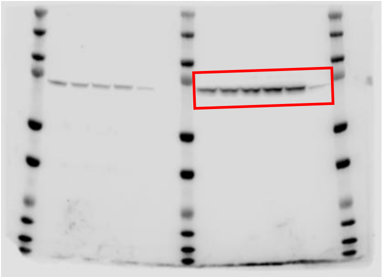

IP: HSP90 (90 kDa)

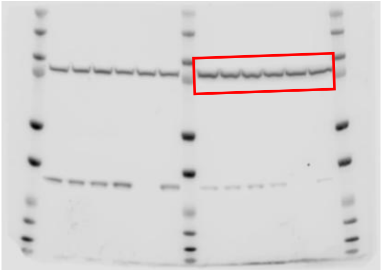

F

IP: ACLY (125 kDa)

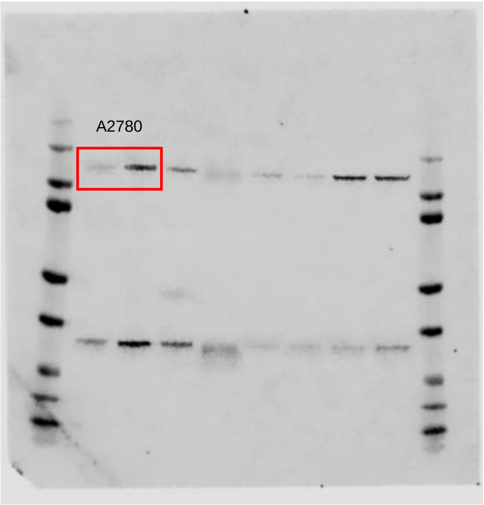

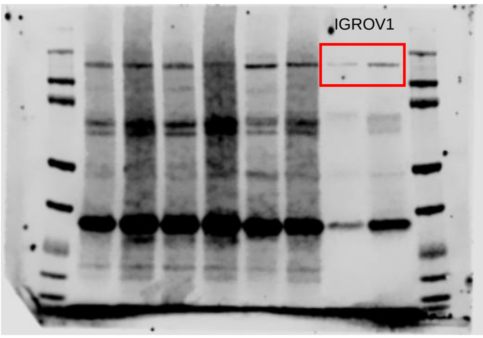

IP: HMGCS1 (57 kDa)

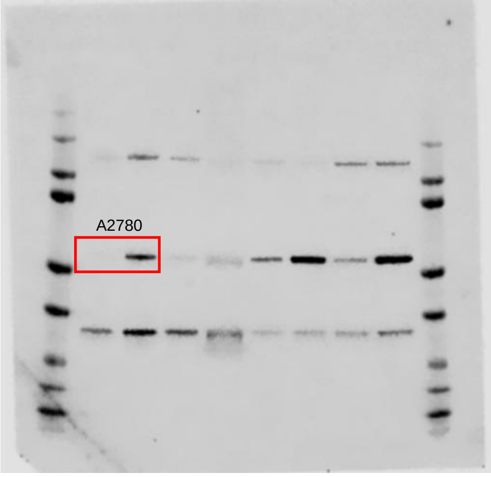

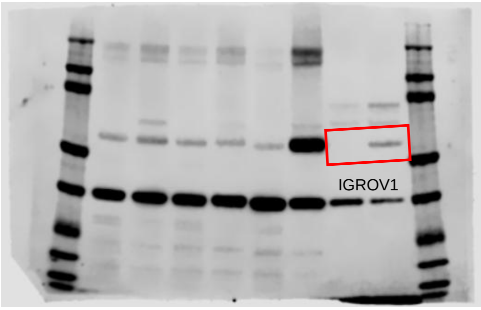

IP: HSP90 (90 kDa)

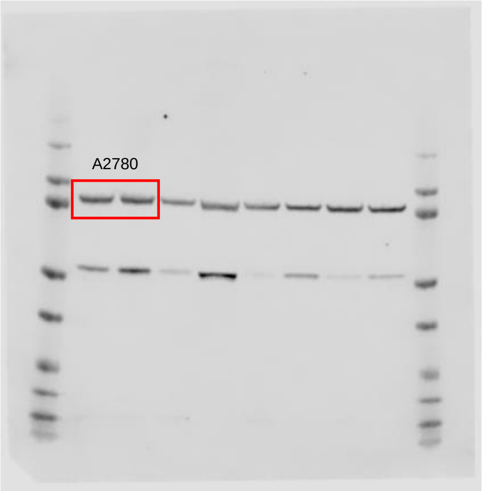

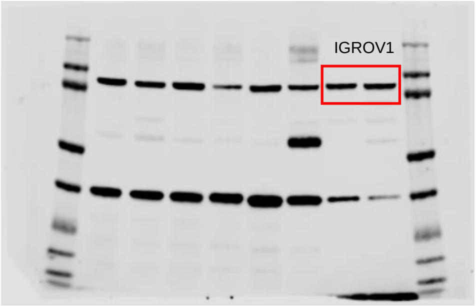

Figure 6

E

IP: PAR

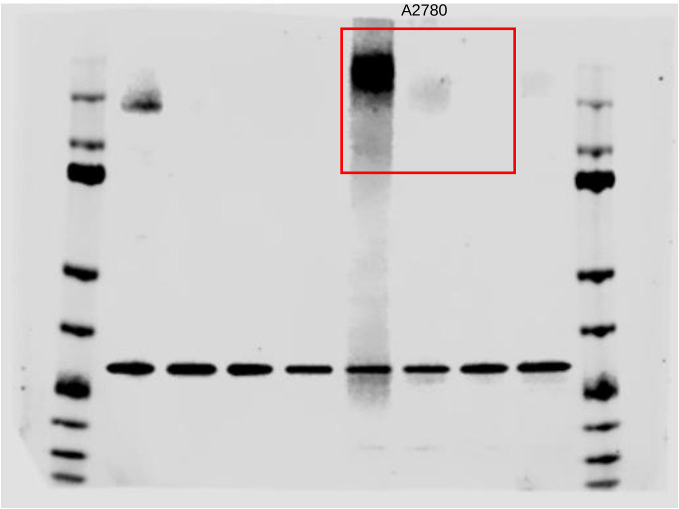

IP: PAR

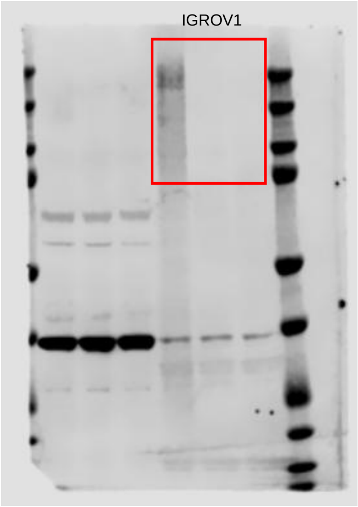

IP: LaminB1 (68 kDa)

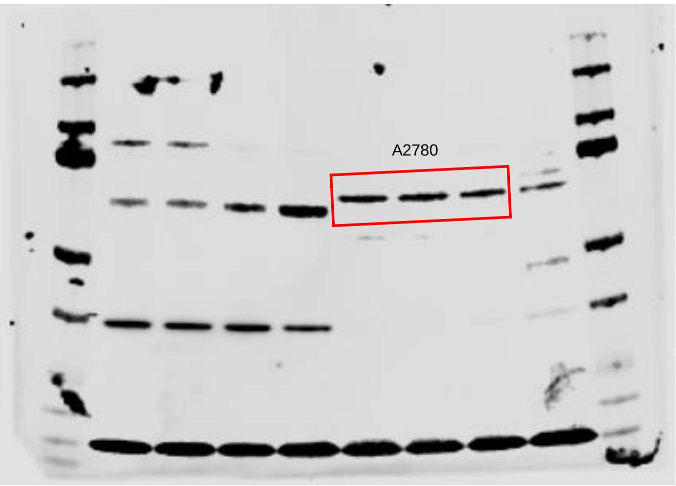

IP: LaminB1 (68 kDa)

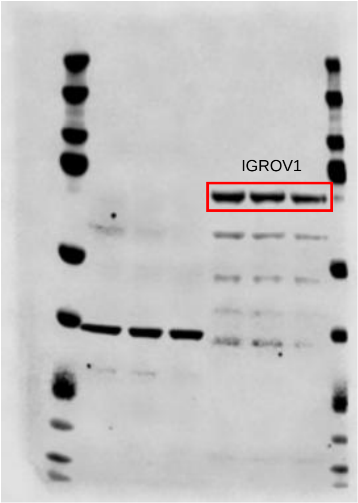

IP: GAPDH (37 kDa)

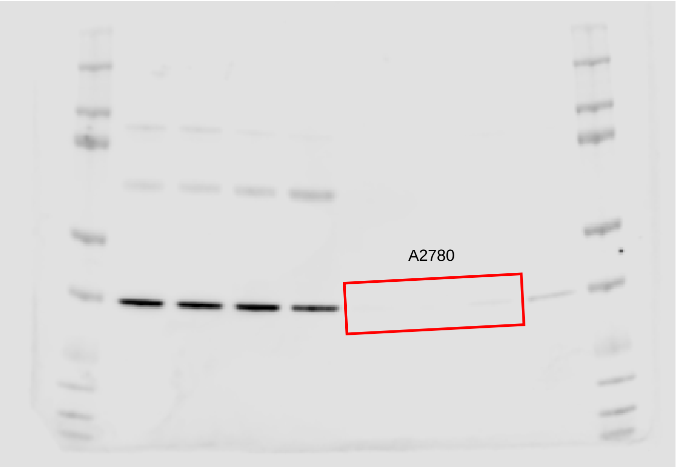

IP: GAPDH (37 kDa)

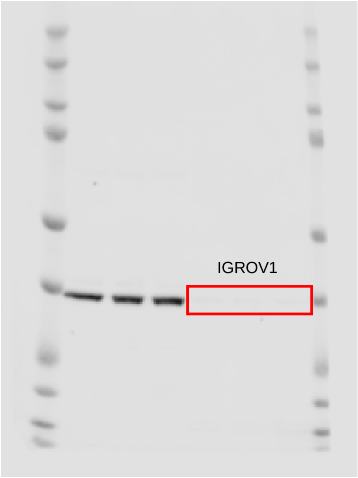

G IP: Cyclin B1 (52 kDa)

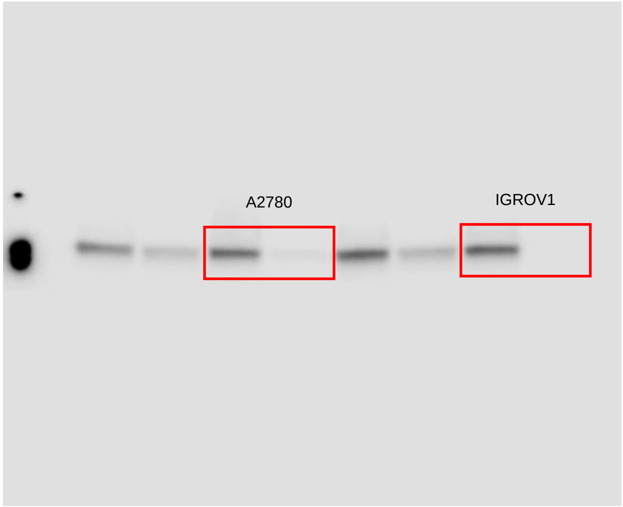

IP: LaminB1 (34 kDa)

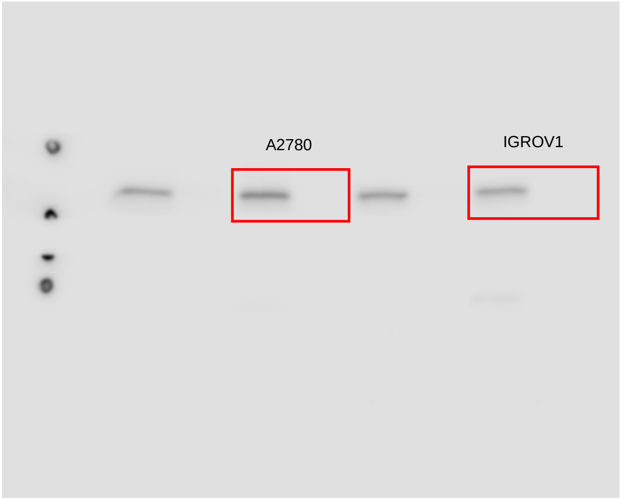

IP: β-actin (42 kDa)

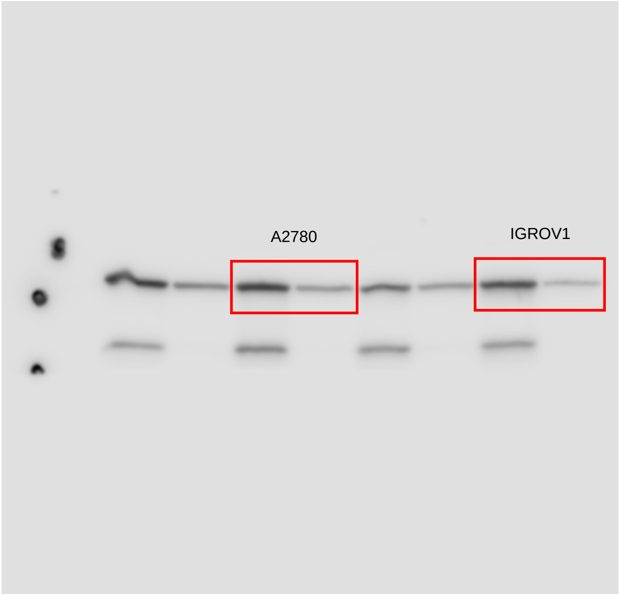

C

IP: CD133 (133kDa)

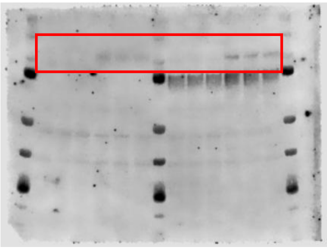

IP: GAPDH (37kDa)

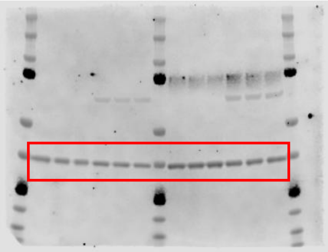

H

IP: NAMPT (52 kDa)

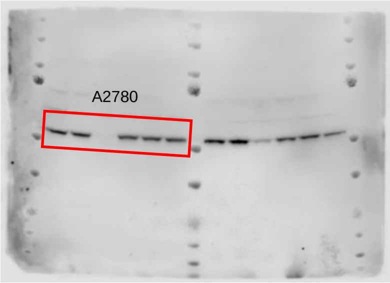

IP: NAMPT (52 kDa)

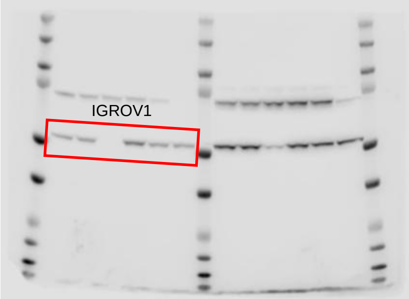

IP: NAPRT (58 kDa)

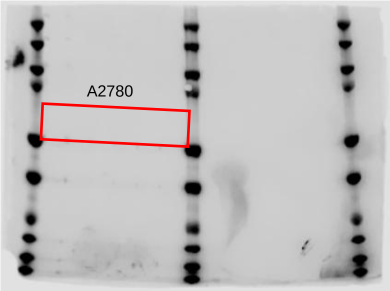

IP: NAPRT (58 kDa)

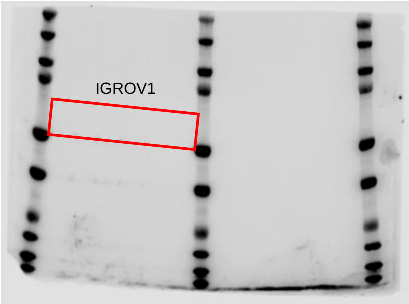

IP: QPRT (31 kDa)

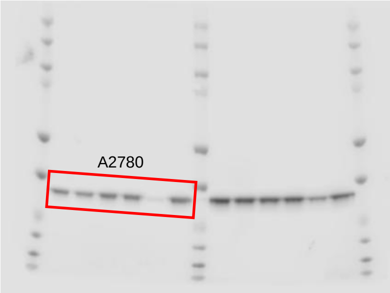

IP: QPRT (31 kDa)

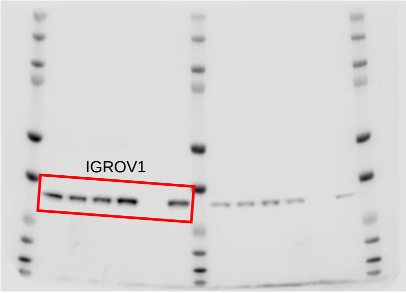

IP: NADSYN1 (79 kDa)

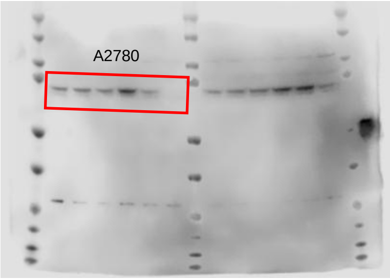

IP: NADSYN1 (79 kDa)

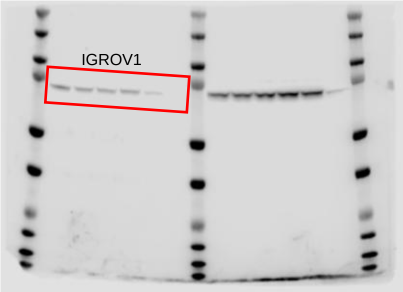

IP: HSP90 (90 kDa)

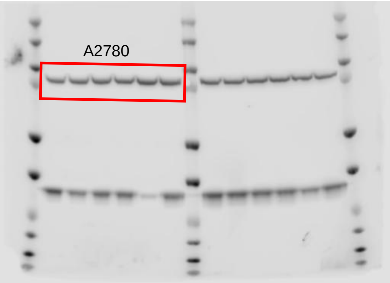

IP: HSP90 (90 kDa)

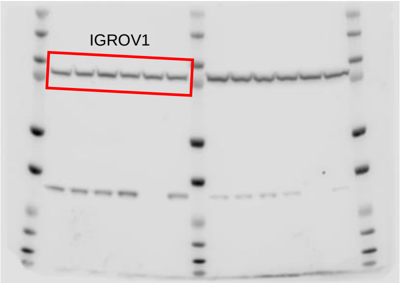

H

IP: Hexokinase II (102 kDa)

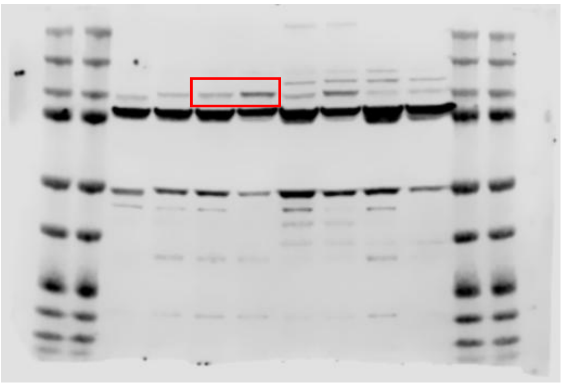

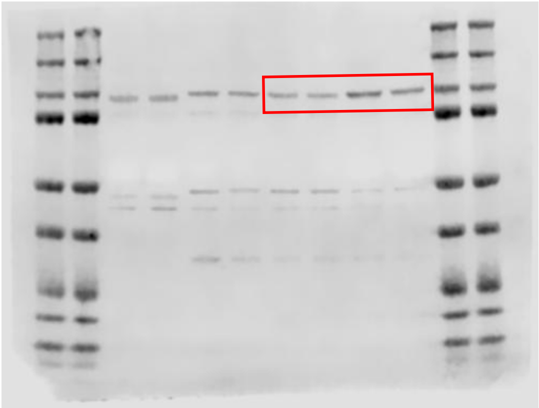

IP: HSP90 (90 kDa)

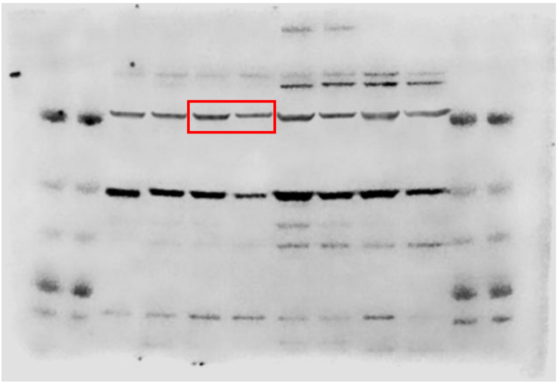

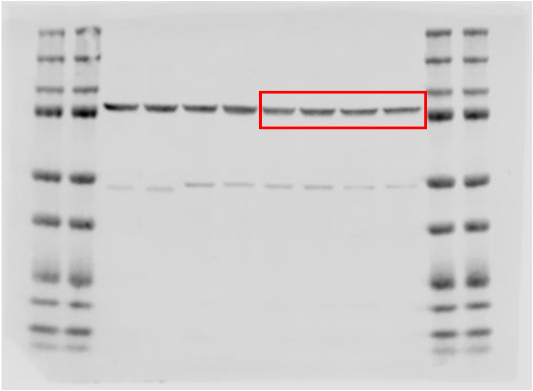

C

IP: Pyruvate carboxylase (130 kDa)

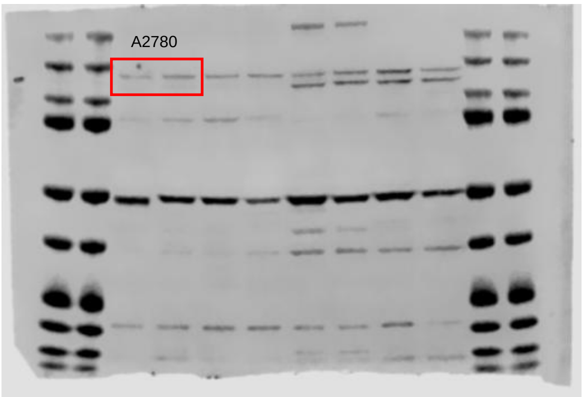

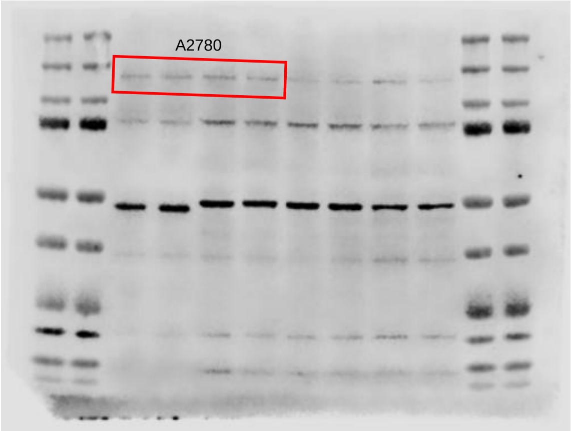

IP: HSP90 (90 kDa)

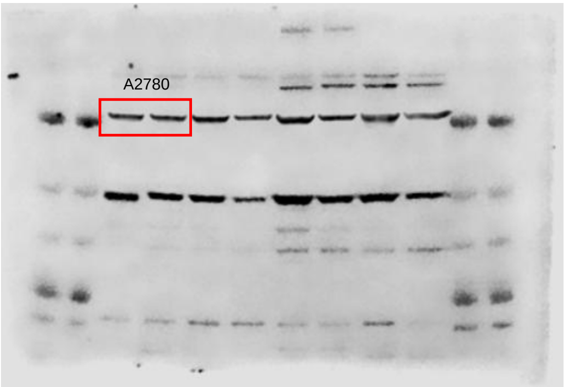

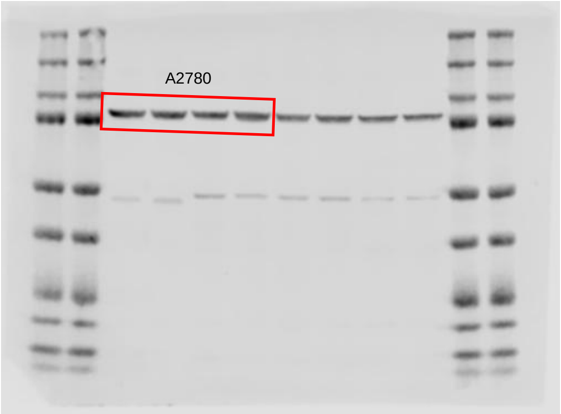

IP: Pyruvate carboxylase (130 kDa)

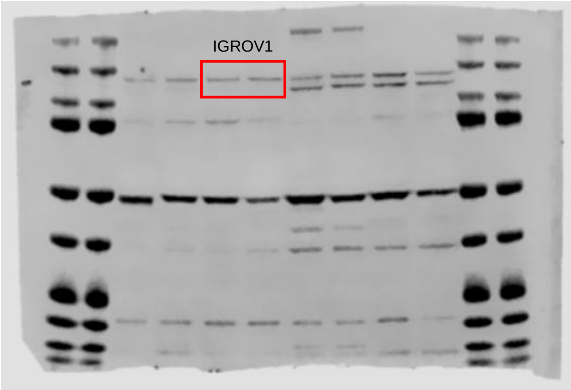

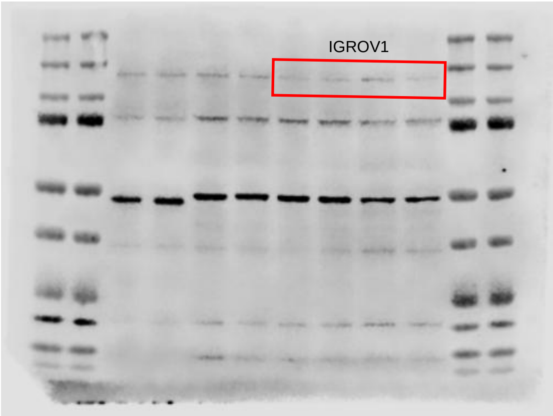

IP: HSP90 (90 kDa)

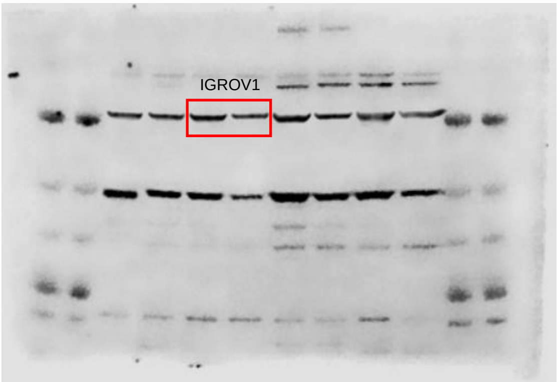

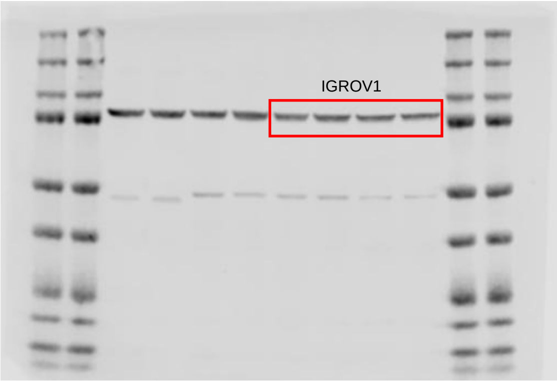

B

IP: Total OXPHOS Human WB Antibody Cocktail

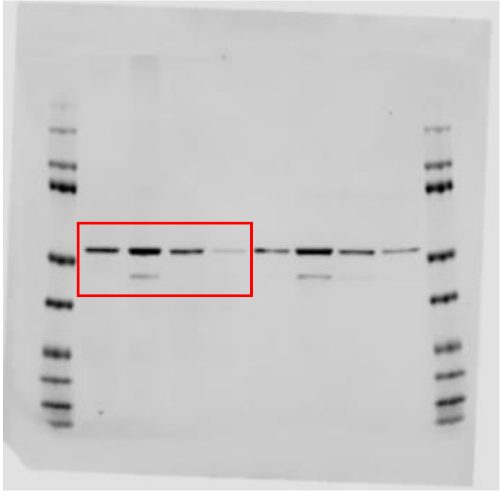

IP: HSP90 (90 kDa)

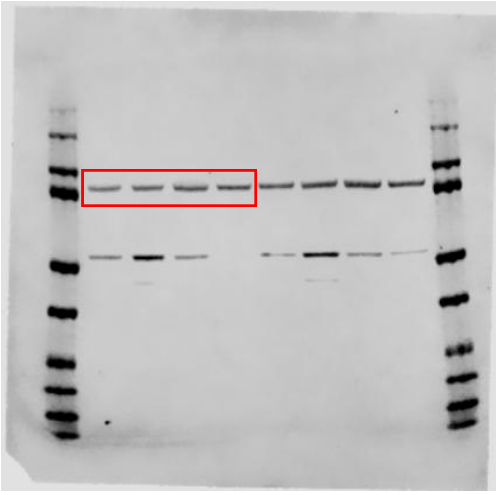

Supplement: Supplementary file 10 — Raw western [file 41419_2025_7672_MOESM10_ESM.pdf]
