# Supplementary material for: Targeting metabolic vulnerability by combining NAMPT inhibitors and disulfiram for treatment of recurrent ovarian cancer
Source: Cell Death Dis. 2025 Apr 25;16(1):342. doi: 10.1038/s41419-025-07672-3 (PMC12032209; doi:10.1038/s41419-025-07672-3)
Supplement: Supplementary file 11 — qPCR primer [file 41419_2025_7672_MOESM11_ESM.docx]

**Reverse transcriptase-PCR**

Primers used for qPCR are listed in the table.

| target gene | GeneGlobe ID | vendor | cat# |
| --- | --- | --- | --- |
| ACTB | QT00095431 | Qiagen | 249900 |
| CD133 | QT00075586 | Qiagen | 249900 |
| CD44 | QT00998333 | Qiagen | 249900 |
| EpCAM | QT00000371 | Qiagen | 249900 |
| KLF4 | QT00061033 | Qiagen | 249900 |
| Myc | QT00035406 | Qiagen | 249900 |
| Nanog | QT01025850 | Qiagen | 249900 |
| SOX2 | QT00237601 | Qiagen | 249900 |
